# Integrated Multi-Omics Analysis Reveals Lipid Metabolism as a Key Contributor to the Growth–Meat Quality Trade-Off Among Genetically Divergent Chicken Breeds

**DOI:** 10.3390/genes17091036

**Published:** 2026-08-29

**Authors:** Ying Li, Rongqin Huang, Li Zhang, Haiping Xu, Chenglong Luo, Wen Luo, Zongliang Du

**Affiliations:** 1 Guangdong Provincial Key Laboratory of Animal Breeding and Nutrition, State Key Laboratory of Swine and Poultry Breeding Industry, Institute of Animal Science, Guangdong Academy of Agricultural Sciences, Guangzhou 510640, China; liying1@gdaas.cn (Y.L.); huangrongqin111@163.com (R.H.); z15001635024@163.com (L.Z.); luochenglong@gdaas.cn (C.L.); duzongliang@gdaas.cn (Z.D.); 2 Key Lab of Chicken Genetics, Breeding and Reproduction, Ministry of Agriculture and Rural Affair, Guangdong Provincial Key Lab of Agro-Animal Genomics and Molecular Breeding, Guangdong Laboratory for Lingnan Modern Agriculture, State Key Laboratory of Swine and Poultry Breeding Industry, South China Agricultural University, Guangzhou 510642, China; 3 School of Food & Pharmaceutical Science and Technology, Guangzhou College of Technology and Business, Guangzhou 510850, China; xuhaiping@gzgs.edu.cn

**Keywords:** meat quality traits, metabolomics, transcriptomics, growth–meat quality trade-off

## Abstract

Background: Improving meat quality while maintaining growth efficiency remains a major challenge in poultry production. However, the molecular mechanisms underlying breed-specific meat quality variation remain unclear. This study aimed to investigate how breed-specific growth patterns influence meat quality and elucidate metabolic and transcriptional mechanisms involved. Methods: Pectoralis major meat quality traits and multi-omics profiles were characterized in three genetically distinct chicken breeds—the fast-growing Small White-Feathered chicken (XBJ), the slow-growing Huiyang Bearded chicken (HXJ), and the layer-type Hy-Line Brown chicken (HLH)—at 50, 180, and 300 days of age. Twelve birds per breed per age were used for phenotypic measurement (*n* = 108 in total), and eight birds per breed per age were subjected to metabolomic and transcriptomic profiling. Phenotypes were analyzed using linear mixed-effects models with breed, age, and their interaction as fixed effects and pen nested within breed as a random effect, followed by Tukey-adjusted pairwise comparisons (*p* < 0.05). Differential metabolites were screened by OPLS-DA (VIP > 1, *p* < 0.05), and differentially expressed genes were identified using DESeq2 (|log2FC| ≥ 1, FDR < 0.05). Integrative analyses were performed to identify key genes, metabolites, and pathways associated with meat quality. Results: Phenotypic evaluation revealed a breed-dependent growth–meat quality trade-off, with XBJ exhibiting superior growth but poorer water-holding capacity and meat color, whereas HXJ and HLH showed better tenderness and color at the expense of growth. Metabolomic analysis revealed lipid metabolism as a major contributor to breed-specific divergence, with triglyceride-driven divergence predominating at early and middle stages, whereas later-stage differences involved glycerophospholipid and amino acid metabolism. Transcriptomic analysis revealed significant breed-specific differences in expressed genes at 50 and 180 days, enriched in pathways related to muscle structure, ECM remodeling, and energy metabolism, consistent with metabolic and phenotypic divergence. Integrated analyses identified 28 candidate genes and 71 core metabolites associated with meat quality traits, with *PLIN1* and *SLC1A6* emerging as key regulators associated with TG species, drip loss, shear force, and BMW. Conclusions: These findings reveal molecular mechanisms underlying the growth–meat quality trade-off and highlight lipid metabolic regulation as a key contributor to meat quality variation. The identified gene–metabolite networks provide insights for molecular breeding to improve chicken meat quality.

## 1. Introduction

Domestic chickens (*Gallus gallus*) are a major global source of animal protein [1]. With the increase in consumer demand for high-quality animal products, improving chicken meat quality has become a central concern in modern poultry production [2]. Meat quality is a complex trait that is typically evaluated through a combination of physical, chemical, and sensory measurements reflecting texture, flavor, and overall eating quality [3,4]. However, the biological mechanisms underlying breed- and age-related differences in chicken meat quality remain poorly understood.

Chicken meat quality is shaped by a complex interplay of factors, with genetic background and age playing key roles, and intensive selective breeding has led to the generation of highly specialized chicken breeds adapted for different production goals, including meat-type, egg-type, and dual-purpose lines [5,6,7]. For instance, modern white-feathered broilers have been intensively selected for rapid growth and high meat yield, but such selection has often been accompanied by reduced sensory quality and metabolic imbalances [8,9,10,11]. In contrast, slow-growing indigenous breeds such as Huiyang Bearded chicken (HXJ) and Beijing-You chicken typically exhibit superior meat quality attributes and richer flavor profiles despite lower production efficiency and reduced yield [12,13,14,15]. Layer-type chickens such as Hy-Line Brown (HLH), primarily selected for egg production, frequently exhibit muscle fiber atrophy and excessive collagen deposition, resulting in tougher meat texture due to reduced selection pressure on breast muscle development [16]. Small White-Feathered chickens (XBJ), a hybrid lineage developed through a three-line breeding system involving white-feathered broilers, indigenous broilers, and egg-laying breeds, including Hy-Line Brown (HLH) as part of the egg-type parental background, have become increasingly popular in China’s broiler market and represent an intermediate production type that balances growth performance and meat quality [17,18].

The selection of these three breeds was motivated by their distinct genetic backgrounds and commercial relevance in China, the world’s second-largest poultry producer. XBJ represents a fast-growing white-feathered broiler hybrid widely used in the southern Chinese market, HXJ is a slow-growing indigenous yellow-feathered breed valued for premium meat quality, and HLH is a globally distributed commercial layer strain. Although HLH is primarily selected for egg production, spent laying hens and culled birds are commonly used for meat in many regions [16], which makes them a relevant comparison for understanding the consequences of divergent selection on muscle biology. Together, these breeds capture a broad spectrum of growth–meat quality strategies and provide a powerful model for dissecting the molecular basis of the growth–meat quality trade-off. However, comparative studies investigating meat quality regulation across these functionally divergent chicken types remain insufficiently explored.

In addition to genetic factors, age represents another key determinant of chicken meat quality [19,20,21]. Previous studies have demonstrated that the physicochemical properties of meat undergo dynamic age-related changes, directly influencing eating quality [21,22]. For example, in the breast muscle of Beijing-You chickens, shear force and intramuscular fat content increase between 90 and 120 days of age, whereas total and essential free amino acid levels decrease [23]. Moreover, significant temporal variations in gene expression patterns and metabolite profiles have been observed in the pectoralis major during the development of yellow-feathered broilers [24]. However, existing studies have largely focused on individual breeds, with limited attention to the dynamic metabolic and transcriptional changes occurring across developmental stages. As a result, the molecular mechanisms driving age-related variation in meat quality remain insufficiently understood [25,26]. Three developmental stages (50, 180, and 300 days) represent key physiological and production phases across different chicken types. At 50 days, XBJ typically reaches its market weight, whereas HXJ and HLH are still in the early growth stages. By 180 days, the slow-growing breed HXJ approaches its market age, whereas the egg-type breed HLH has largely completed muscle growth, with metabolism increasingly oriented toward supporting egg production. At 300 days, HLH is near the end of its productive lifespan, with physiological and metabolic characteristics significantly influenced by prolonged egg production. Although transcriptomic and metabolomic techniques have been increasingly applied in poultry research, comprehensive multi-omics studies integrating gene expression and metabolic processes across genetically distinct chicken breeds and developmental stages to elucidate the growth–meat quality trade-off remain limited.

To address this gap, this study aimed to investigate how breed-specific growth patterns influence meat quality traits across different developmental stages and to elucidate the metabolic and transcriptional processes mediating these differences. A comparative analysis of growth and meat quality traits in the pectoralis major of three genetically distinct chicken breeds, including the fast-growing XBJ, the slow-growing HXJ, and the layer-type HLH, across three developmental stages (50, 180, and 300 days) was performed. Breed- and age-dependent differences in gene expression and metabolite profiles were systematically characterized using transcriptomic and metabolomic approaches, respectively. Correlation analyses were further conducted to identify key regulatory genes and metabolites associated with meat quality traits, as well as the metabolic pathways underlying these associations, providing the basis for the construction of gene–metabolite regulatory networks involved in the growth–meat quality trade-off. This multi-omics framework reveals how lipid metabolism, energy regulation, and muscle development interact to shape breed-specific differences in critical traits such as drip loss, shear force, and muscle color. This study provides a comprehensive molecular perspective on the regulation of meat quality in poultry and offers valuable insights for optimizing breeding strategies to improve meat quality without compromising growth performance.

## 2. Materials and Methods

### 2.1. Animals and Sample Collection

A total of 450 one-day-old female chicks were used in this study, with 150 chicks allocated to each of the three genetically distinct breeds: the fast-growing Small White-Feathered chicken (XBJ), the slow-growing Huiyang Bearded chicken (HXJ), and the Hy-Line Brown layer chicken (HLH). All chicks were sourced from standardized large-scale breeding farms with equivalent health management protocols and were certified as free from major avian diseases. To minimize the confounding effects of feed nutrition, housing environment, and management practices on meat quality, the three breeds were reared under standardized conditions within the same poultry house but in separate pens. Chicks from each breed were randomly assigned to 5 floor pens (30 birds per pen) within the same poultry house. Birds were reared under standardized management conditions with rice hull bedding, natural lighting, and natural ventilation. All birds had ad libitum access to pelleted feed and water throughout the experimental period. Although the three breeds have different optimal management recommendations under commercial production, the objective of this study was to compare their genetic responses under a standardized environment. Therefore, identical commercial diets, ventilation, and health management protocols were applied across all pens. Three calendar-based feeding phases were adopted according to the commercial feed specifications: starter diets (0–30 days), grower diets (31–60 days), and finisher diets (61–300 days). Diets were formulated to meet NRC (1994) minimum nutrient requirements for poultry and supplied by Guangdong Guangda Biological Nutrition Technology Co., Ltd., Jiangmen, China. The analyzed nutrient compositions of the experimental diets are provided in Table 1. These feeding phases were defined by chronological age rather than breed-specific market or physiological ages. Nevertheless, all three breeds received identical diets at the same chronological age throughout the experiment, eliminating diet as a confounding factor for cross-breed comparison. Routine husbandry and vaccination were applied according to standard protocols. Each bird was individually identified using uniquely numbered wing bands corresponding to its breed, and detailed records were maintained throughout the trial. All animal experiments were conducted in accordance with the protocols approved by the Animal Care Committee of the Institute of Animal Science, Guangdong Academy of Agricultural Sciences, Guangzhou, P. R. China, with the approval number GAASIAS-2023015.

At each designated time point (50, 180, and 300 days of age), 12 healthy birds per breed were randomly selected from different pens and slaughtered for growth and meat quality evaluation and sample collection. Prior to slaughter, birds were fasted for 12 h with continuous access to water. Body weights were recorded, and birds were then humanely euthanized following approved protocols. Both pectoralis major muscles were carefully excised. The left pectoralis major was weighed and used for comprehensive meat quality assessments, while the right pectoralis major was flash-frozen in liquid nitrogen and stored at −80 °C for subsequent metabolomic and transcriptomic analyses.

### 2.2. Meat Quality Characteristics

Slaughter and Carcass Processing. At each sampling age, birds were fasted for 12 h with free access to water and weighed individually. All birds were electrically stunned and then humanely slaughtered via cervical dislocation following standard poultry slaughter procedures. After complete exsanguination, carcasses were scalded, defeathered, and eviscerated. The left pectoralis major muscle was excised for meat quality determination, while the right pectoralis major was immediately snap-frozen in liquid nitrogen and stored at −80 °C for subsequent transcriptomic and metabolomic analyses. Meat quality traits were measured using standard procedures as described previously [27,28,29].

pH. Muscle pH was measured using a pH-STAB meter (Matthãus, Berlin, Germany), calibrated with standard buffer solutions (pH 7.00 and pH 4.64) prior to measurement. Three readings were taken from different sites within the central region of the pectoralis major, and the mean value was calculated.

Meat Color. Meat color parameters—lightness (L*), redness (a*), and yellowness (b*)—were measured using a 3nH-NH310 colorimeter (3NH, Guangzhou, China). The instrument was calibrated with a standard white tile according to the manufacturer’s instructions. Before measurement, surface connective tissue was carefully removed, and samples were placed on a flat surface. The colorimeter probe was positioned perpendicular to the measurement site, and three readings per sample were averaged.

Shear Force. Meat tenderness was evaluated using a texture analyzer (Model C-LM3B, BeiJing Tianxiang Feiyu Technology Co., Ltd., Beijing, China) equipped with a Warner–Bratzler shear blade. Subsamples (1 cm × 1 cm × 3 cm) were prepared from the cranial, middle, and caudal regions of the left pectoralis major, respectively, parallel to the muscle fiber orientation. Each sample was sheared perpendicular to the fiber. Three replicates were measured per muscle, and the mean value was used for statistical analysis.

Drip Loss. Excess fat and connective tissue were trimmed from the pectoralis major, and 10 g samples were collected. Each sample was weighed, suspended vertically in an inflated polyethylene bag using a wire to prevent contact with the bag walls, and stored at 4 °C for 24 h. After storage, samples were reweighed, and drip loss (%) was calculated as: [initial weight of the meat sample (g)—post-storage weight of the meat sample (g)]/initial weight of the meat sample (g) × 100.

### 2.3. Preparation and Extraction of Metabolomic Samples

Sample Extraction. For untargeted metabolomic analysis, eight birds per breed and age group (50, 180, and 300 days) were selected based on pectoralis major weights closest to the respective group mean, with birds distributed across the five replicate pens. Sample preparation and extraction were performed following established protocols described by previous researchers [30]. Firstly, samples were retrieved from the −80 °C freezer and thawed on ice until they could be sectioned (all subsequent operations were conducted on ice). The thawed tissue was finely chopped and mixed. Then 20 mg (±1 mg) of tissue was placed into pre-labeled centrifuge tubes. A steel bead was added using tweezers, and the tissue was homogenized using a ball mill at 30 Hz for 20 s. The homogenate was briefly centrifuged at 3000 rpm for 30 s at 4 °C, after which 400 μL of 70% methanol containing internal standards was added. The mixture was vortexed at 1500 rpm for 5 min, incubated on ice for 15 min, and then centrifuged at 12,000 rpm for 10 min at 4 °C. Subsequently, 300 μL of the supernatant was transferred to a new centrifuge tube, stored at −20 °C for 30 min, and centrifuged again at 12,000 rpm for 3 min at 4 °C. Finally, 200 μL of the clarified supernatant was transferred into a sample vial with a liner insert for subsequent LC-MS analysis.

LC-MS/MS Analysis and Metabolite Identification. After extraction, metabolite profiling was conducted using a UPLC-ESI-MS/MS system (ExionLC™ AD ultra-performance liquid chromatograph coupled with a QTRAP^®^ mass spectrometer, SCIEX, Framingham, MA, USA). Hydrophilic metabolites were separated on a Waters HSS T3 C18 column (1.8 µm, 2.1 × 100 mm, Waters, Milford, MA, USA) with a mobile phase consisting of water + 0.1% formic acid and acetonitrile using a 14 min gradient program. Hydrophobic metabolites were separated on a Thermo C30 column (2.6 µm, 2.1 × 100 mm, Thermo Fisher Scientific, Waltham, MA, USA) with a biphasic solvent system of acetonitrile/water and acetonitrile/isopropanol. Mass spectrometry was performed in both positive and negative electrospray ionization (ESI) modes, with a source temperature of 500 °C, spray voltage of ±5500 V, and ion source gas pressures ranging from 45 to 60 psi. Data acquisition was performed in multiple reaction monitoring (MRM) mode. Metabolites were identified and quantified using a self-constructed MetWare database (MWDB) based on the alignment of retention times and fragmentation patterns.

### 2.4. Quality Control and Differential Metabolite Analysis

To ensure data reliability, pooled quality control (QC) samples were utilized to monitor instrument performance. Only metabolites with a coefficient of variation (CV) ≤ 15% in QC samples were retained for downstream analysis. Data were normalized and scaled by unit variance. Multivariate statistical analyses were conducted in R, and differential metabolites were identified using variable importance in projection (VIP) > 1 from orthogonal partial least squares discriminant analysis (OPLS-DA) combined with Student’s *t*-test (*p* < 0.05) for pairwise breed comparisons within each developmental stage or one-way ANOVA (*p* < 0.05) for comparisons among multiple groups. Volcano plots visualized significant changes, with log_2_ fold change on the x-axis and –log_10_(*p*-value) on the y-axis, and upregulated or downregulated genes highlighted by distinct colors.

### 2.5. RNA Extraction and Transcriptomic Analysis

RNA Extraction. Pectoralis major samples were pulverized in liquid nitrogen. Total RNA was extracted using TRIzol™ Reagent (Invitrogen, Carlsbad, CA, USA) at 1 mL per 50 mg of tissue. Homogenization was performed with a mechanical homogenizer following the manufacturer’s instructions. RNA concentration and integrity were assessed using a NanoDrop™ ND-1000 spectrophotometer (NanoDrop Technologies, Wilmington, DE, USA). Poly(A) mRNA was enriched using oligo(dT) magnetic beads, followed by random fragmentation in a fragmentation buffer containing divalent cations at elevated temperature. The fragmented mRNA was used as a template for first-strand cDNA synthesis primed with random hexamers, after which the RNA template was degraded with RNase H, and second-strand cDNA synthesis was performed using DNA Polymerase I and dNTPs. The resulting double-stranded cDNA was purified, end-repaired, A-tailed, and adapter-ligated. The ligated products were size-selected using AMPure XP beads (Beckman Coulter, Brea, CA, USA) to obtain fragments in a range of 370–420 bp. These size-selected fragments were then PCR-amplified with index primers, purified again using AMPure XP beads, and finally quantified and quality-controlled for sequencing.

Data Processing. Sequencing was performed on the Illumina NovaSeq platform (Illumina, Inc., San Diego, CA, USA), generating 150 bp paired-end reads. Raw reads were processed using fastp (V0.23.2, https://github.com/OpenGene/fastp (accessed on 29 July 2026)); reads containing adapter sequences were removed, and those containing more than 10% ambiguous bases (N) or with low-quality bases (Q ≤ 20) that accounted for over 50% of the total bases were discarded. The resulting clean reads were aligned to the reference genome (Gallus_gallus.bGalGal1.mat.broiler.GRCg7b.DNA.toplevel.fa.gz) using HISAT2 (V2.2.1, http://daehwankimlab.github.io/hisat2/ (accessed on 29 July 2026)), and novel gene prediction was conducted with StringTie (version 1.3.3b, https://ccb.jhu.edu/software/stringtie/ (accessed on 29 July 2026)). Read counts were obtained with FeatureCounts (V1.5.0-p3, http://subread.sourceforge.net/ (accessed on 29 July 2026)), and fragments per kilobase of transcript per million mapped reads (FPKM) values were calculated based on gene length and the number of aligned reads. 

Differential Expression Analysis and Functional Annotation. Differentially expressed genes (DEGs) were identified using the DESeq2 package (V1.34.0, https://bioconductor.org/packages/release/bioc/html/DESeq2.html (accessed on 29 July 2026)), which applies a model based on the negative binomial distribution. The Benjamini–Hochberg (BH) method was used to adjust *p*-values for multiple testing to control the false discovery rate (FDR). Genes were defined as differentially expressed with the criteria of |log_2_(fold change)| ≥ 1 and FDR < 0.05. Gene Ontology (GO) and Kyoto Encyclopedia of Genes and Genomes (KEGG) enrichment were conducted using the ClusterProfiler package (V4.2.2, https://bioconductor.org/packages/release/bioc/html/clusterProfiler.html (accessed on 29 July 2026)), and the results were visualized with the ggplot2 package in R (V3.4.2, https://ggplot2.tidyverse.org/). Gene interaction networks were explored via STRING (https://cn.string-db.org/, accessed on 15 December 2024), and visualized using Cytoscape (V3.10.0, https://cytoscape.org/).

### 2.6. Statistical Analysis

For growth performance and meat quality traits, linear mixed-effects models (LMMs) were fitted using the lme4 package (V2.0-6, https://cran.r-project.org/package=lme4 (accessed on 29 July 2026)) and the lmerTest package (V3.2-1, https://cran.r-project.org/package=lmerTest (accessed on 29 July 2026)) in R. The model included breed, age, and their two-way interaction as fixed effects, and pen nested within breed as a random intercept (Trait~Breed × Age + (1|Breed:Pen) + residual). Pairwise comparisons among breeds within each age were based on estimated marginal means with Tukey’s adjustment using the emmeans package (V2.0.4, https://cran.r-project.org/package=emmeans (accessed on 29 July 2026)). Pen effects and pen × breed interactions were evaluated and are reported in Supplementary Table S1. Additionally, sensitivity analyses including body weight as a covariate were performed for meat quality traits. A threshold of *p* < 0.05 was considered significant.

## 3. Results

### 3.1. Phenotypic Analysis Reveals a Trade-Off Between Growth Efficiency and Meat Quality in Different Breeds

This study assessed the breast meat quality and growth performance of three genetically distinct chicken breeds: the fast-growing XBJ (market age: 50 days), the slow-growing, high-quality HXJ (market age: 120–180 days), and the dual-purpose HLH (primarily for egg production, with meat as a byproduct). Their growth and quality profiles were consistent with their respective breeding goals.

**Figure 1:**
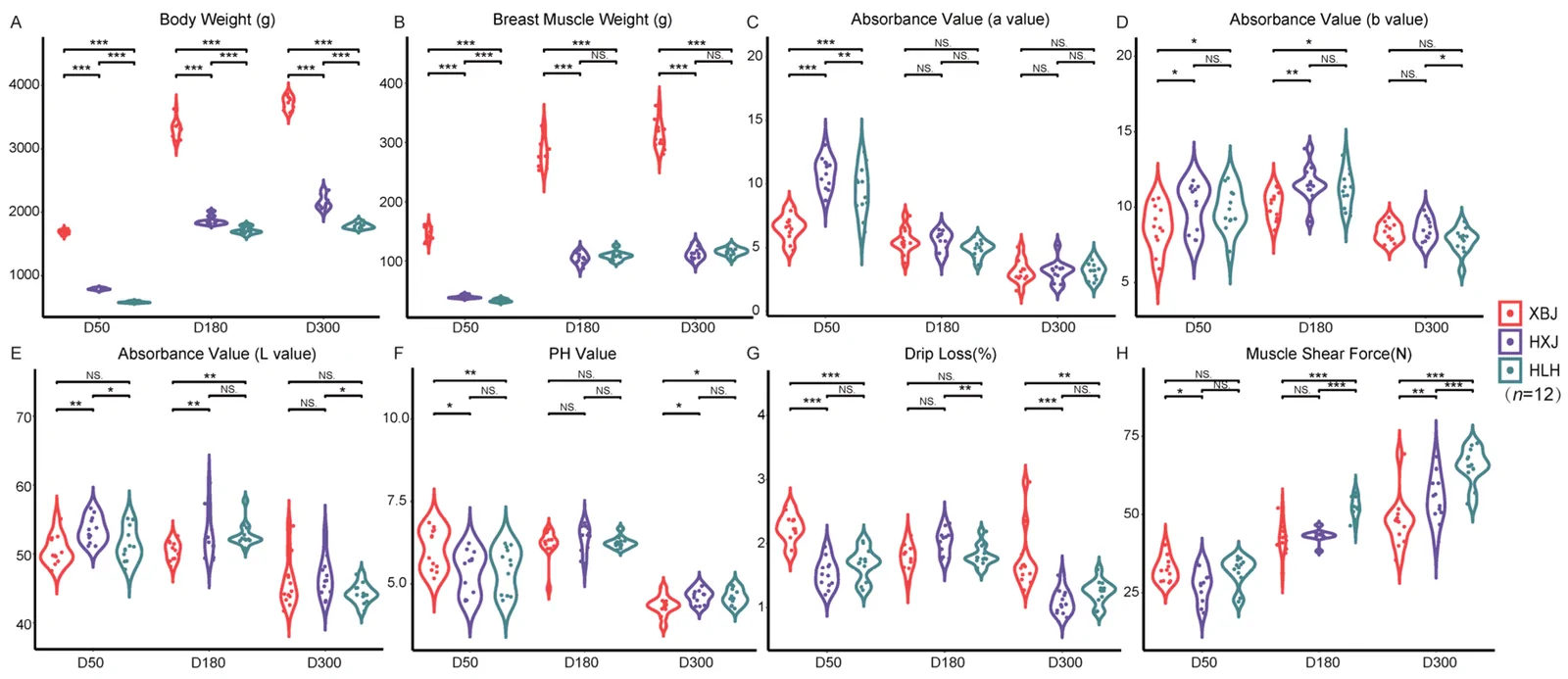
Meat quality characteristics of the breast muscle of the Small White-feather chicken, Huiyang Bearded chicken and Hy-Line Brown chicken. (**A**) Live weight of the three chicken breeds at different ages. (**B**) Breast muscle weight of the three chicken breeds at different growth ages. (**C**) Redness, (**D**) yellowness, (**E**) lightness, (**F**) pH, (**G**) drip loss, and (**H**) shear force of the breast muscle at different growth ages for the three chicken breeds. Statistical comparisons were based on linear mixed-effects models with Tukey’s adjustment. * *p* < 0.05, ** *p* < 0.01, *** *p* < 0.001, NS indicates *p* > 0.05.

As expected, XBJ exhibited a significant growth advantage, with both body weight and breast muscle weight consistently higher than those of HXJ and HLH (*p* < 0.001) throughout the entire growth cycle (Figure 1A,B). By day 300, the average body weight of XBJ chickens reached approximately 4000 g, significantly surpassing the other two breeds (*p* < 0.001). These results confirmed XBJ’s genetic potential for efficient lean tissue accretion, which was consistent with its selective breeding for rapid growth.

Analysis of meat quality found common trends across all three breeds, including a decrease in redness (a*), lightness (L*), and pH with age, while shear force values increased, reflecting the influence of age on meat quality development. On the other hand, pronounced meat quality differences were observed among XBJ, HXJ, and HLH. At its market age of 50 days, XBJ showed significantly lower redness (a*) than HXJ (*p* < 0.001) and HLH (*p* < 0.001), as well as significantly lower yellowness (b*) than both HXJ and HLH (*p* < 0.05), and lower lightness (L*) than HLH (*p* < 0.01) (Figure 1C–E). These findings reflected its selection for rapid growth efficiency at the expense of favorable color attributes. In contrast, HXJ and HLH at 180 days exhibited more desirable meat color, with significantly higher yellowness (b*) than XBJ (*p* < 0.05), whereas lightness (L*) did not differ significantly among breeds at this age (Figure 1D,E). At the same time, water-holding capacity (WHC) also diverged markedly among XBJ, HXJ and HLH. XBJ showed the highest drip loss at both 50 and 300 days (*p* < 0.001), confirming its consistently poor WHC, which is a common trade-off in fast-growing breeds (Figure 1G). Interestingly, HXJ showed a marked peak in drip loss at 180 days; however, this peak did not reach statistical significance compared with XBJ or HLH after accounting for pen-level variation (*p* > 0.05). By contrast, HLH consistently maintained relatively low drip loss throughout the entire growth cycle, suggesting a more stable water retention across ages.

Age-related dynamics in pH, drip loss, and shear force revealed distinct post-mortem muscle characteristics among XBJ, HXJ, and HLH. XBJ showed the highest pH at 50 days, significantly higher than both HXJ and HLH (*p* < 0.01), but pH differences among breeds were not significant by 300 days (*p* > 0.05) (Figure 1F), accompanied by persistently high drip loss at both ages (Figure 1G). These opposing shifts in pH and WHC highlighted breed-specific post-mortem metabolic dynamics in XBJ muscle that compromised water retention. Although shear force increased with age in XBJ, HXJ and HLH (Figure 1H), the relative patterns varied. At 50 days, shear force did not differ significantly among the three breeds (*p* > 0.05). At 180 days, XBJ showed significantly lower shear force than HXJ (*p* < 0.001) but was comparable to HLH, whereas HXJ exhibited significantly higher shear force than HLH (*p* < 0.01). These differences became more pronounced with age. By 300 days, XBJ showed the lowest shear force among the three breeds (*p* < 0.001 compared to HXJ and *p* < 0.01 compared to HLH). These results indicated that although XBJ followed the general trend toward tougher meat with age, its muscle remained relatively more tender at later stages, suggesting intrinsic structural characteristics that differentiated it from HXJ and HLH. In contrast, HLH consistently produced the toughest meat, particularly at D300 (*p* < 0.001), and showed significantly higher shear force than HXJ at D180 (*p* < 0.01), reflecting muscle characteristics optimized for endurance to support its primary role in prolonged egg production rather than meat tenderness. Overall, these phenotypic results revealed a clear trade-off among the three breeds and provided a basis for subsequent multi-omics analyses to elucidate the molecular mechanisms underlying this trade-off.

### 3.2. Metabolomic Profiling Reveals Breed-Specific Lipid Differences and Highlights Triglycerides as Key Determinants of Meat Quality Divergence

**Figure 2:**
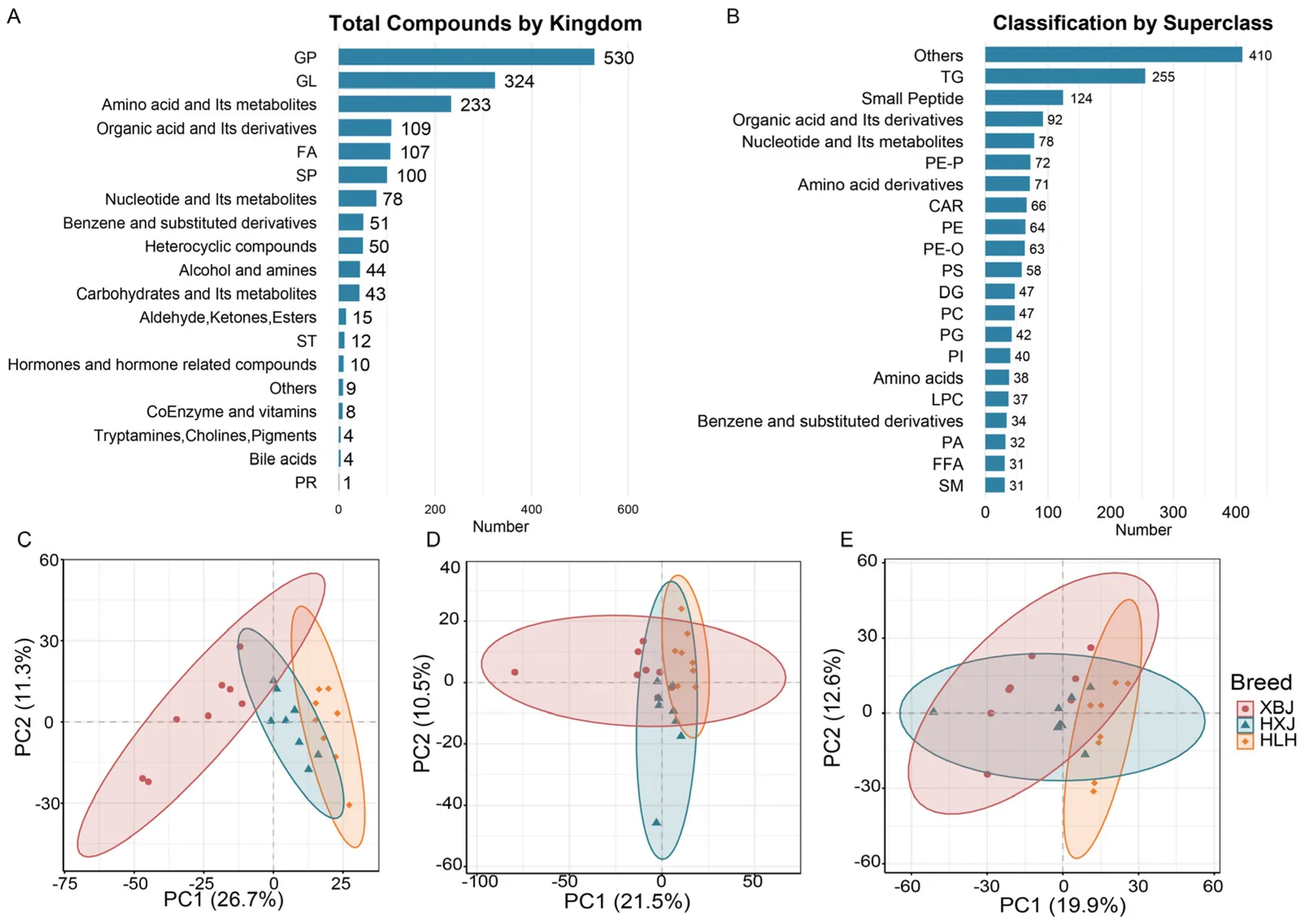
Metabolic profiling of pectoralis major across developmental stages in three chicken breeds. (**A**) Relative abundance of metabolite classes at the Kingdom level, presented as a horizontal bar graph. (**B**) Relative abundance of the top 20 metabolite superclasses; all remaining low-abundance superclasses were aggregated into the “Others” category. (**C**–**E**) PCA score plots of metabolites identified in three chicken breeds at 50 days of age (**C**), 180 days of age (**D**), and 300 days of age (**E**). *n* = 8 for omics analyses.

LC-MS-based metabolomic profiling of breast muscle was performed to explore the metabolic basis for phenotypic differences among breeds. A total of 1732 metabolites were identified (1067 in positive mode and 665 in negative mode), with lipids as the predominant class (Figure 2A). Glycerophospholipids (GP, 30.6%) and glycerolipids (GL, 18.71%) were the most abundant categories, with triglycerides (TGs) as the dominant subclass (14.72%) (Figure 2A,B), indicating lipid metabolism as a major contributor to breed-specific variation in meat quality.

**Figure 3:**
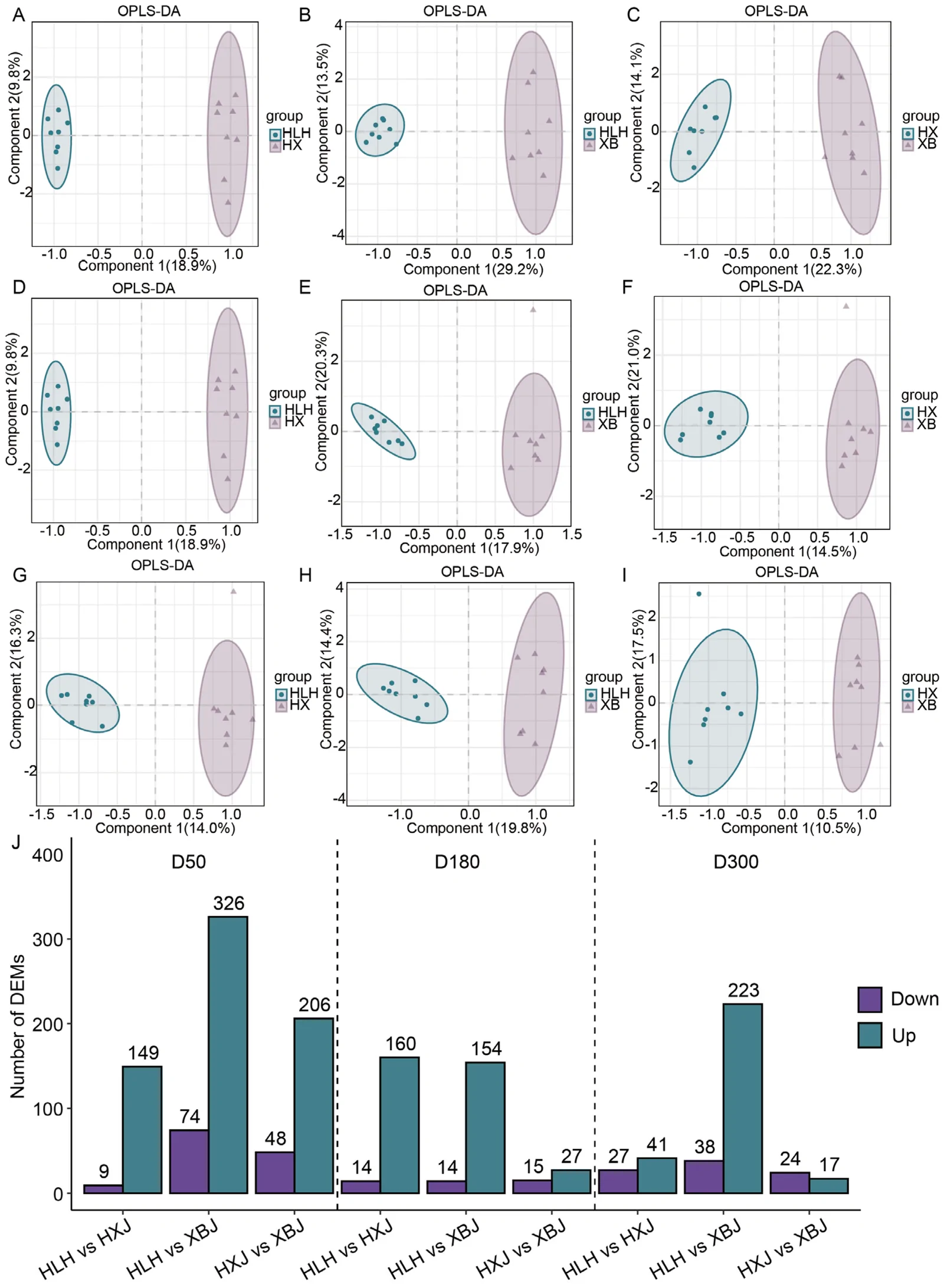
Differentially expressed metabolites in the pectoralis major of three chicken breeds. (**A**–**C**) OPLS-DA scores of the identified metabolites in the pectoralis major of HXJ vs. HLH (**A**), XBJ vs. HLH (**B**), and HXJ vs. XBJ (**C**) at 50 days of age. (**D**–**F**) OPLS-DA scores of the identified metabolites in the pectoralis major of HXJ vs. HLH (**D**), XBJ vs. HLH (**E**), and HXJ vs. XBJ (**F**) at 180 days of age. (**G**–**I**) OPLS-DA scores of the identified metabolites in the pectoralis major of HXJ vs. HLH (**G**), XBJ vs. HLH (**H**), and HXJ vs. XBJ (**I**) at 300 days of age. (**J**) Histogram of the number of differential metabolites among different groups. *n* = 8 for omics analyses.

To further examine breed-specific metabolic variation, multivariate analyses were performed on pectoralis major samples. Principal component analysis (PCA) revealed clear separation of XBJ, HXJ, and HLH at each time point (Figure 2C–E). Orthogonal partial least squares-discriminant analysis (OPLS-DA) further demonstrated breed-specific metabolic signatures with robust model performance (R^2^Y > 0.9, Q^2^ > 0.5) across all sampling points (Figure 3A–I), suggesting high reliability for subsequent downstream analyses (Figure S1). These results indicated that breed-specific adjustments in metabolic pathways contributed to the phenotypic differences observed in growth and meat quality.

**Figure 4:**
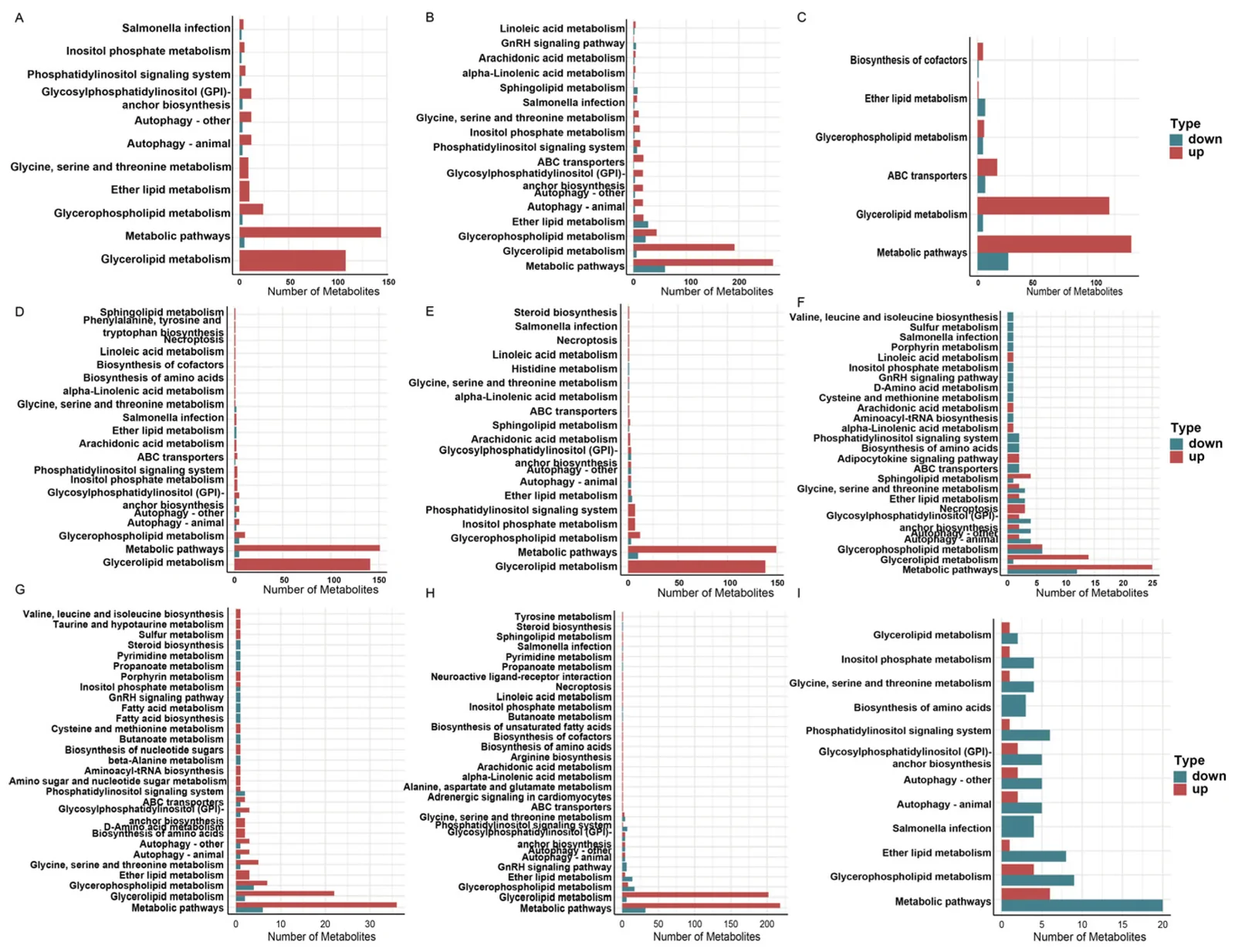
KEGG enrichment analysis of differential metabolites in the pectoralis major of three chicken breeds. (**A**–**C**) KEGG enrichment pathways of differential metabolites in the pectoralis major of HXJ vs. HLH (**A**), XBJ vs. HLH (**B**), and HXJ vs. XBJ (**C**) at 50 days of age. (**D**–**F**) KEGG enrichment pathways of differential metabolites in the pectoralis major of HXJ vs. HLH (**D**), XBJ vs. HLH (**E**), and HXJ vs. XBJ (**F**) at 180 days of age. (**G**–**I**) KEGG enrichment pathways of differential metabolites in the pectoralis major of HXJ vs. HLH (**G**), XBJ vs. HLH (**H**), and HXJ vs. XBJ (**I**) at 300 days of age. *n* = 8 for omics analyses.

Differentially expressed metabolites (DEMs) were identified among the three breeds using thresholds of VIP > 1 and *p* < 0.05. At 50 days, 254 and 400 DEMs were detected in XBJ compared with HXJ and HLH, respectively, whereas 158 DEMs were identified between HXJ and HLH (Figure 3J). At 180 days, 42 and 168 DEMs were identified in XBJ compared with HXJ and HLH, respectively, whereas 174 were detected between HXJ and HLH (Figure 3J). By D300, the metabolic differences between XBJ and HXJ or between HXJ and HLH were relatively minor, whereas 261 DEMs were detected between XBJ and HLH (223 upregulated in XBJ) (Figure 3J). DEM classification analysis revealed TGs as the predominant differential metabolite class across breeds at multiple stages. At 50 days, XBJ exhibited markedly elevated TGs compared with both HLH and HXJ, consistent with its rapid growth and energy storage demands (Figure S2A,B). HXJ also displayed 105 upregulated TGs compared with HLH (Figure S2C). Pathway enrichment analysis revealed that most of these TGs were enriched in glycerolipid metabolism and fatty acid biosynthesis (Figure 4A–C), further highlighting enhanced lipid deposition and energy storage. At 180 days, TG accumulation remained prominent in HLH comparisons, with HXJ and XBJ showing 140 and 132 upregulated TGs, respectively. In contrast, differences between HXJ and XBJ shifted toward glycerophospholipid (GP) metabolism, while HXJ and XBJ exhibited comparable numbers of GPs, although XBJ still displayed higher TG abundance (Figure S2D–F). By 300 days, although TGs remained predominant in HLH comparisons with HXJ and XBJ, their numbers declined markedly compared with earlier stages (22 and 29 upregulated TGs, respectively). Furthermore, the HXJ vs. XBJ comparison was characterized primarily by differences in glycerophospholipids and fatty acids rather than TGs (Figure S2G–I). While enrichment analysis of HLH comparisons (HXJ vs. HLH and XBJ vs. HLH) consistently identified metabolic pathways and glycerolipid metabolism as the top categories across all ages, HXJ vs. XBJ displayed a more diversified enrichment pattern at D180 and D300, involving glycerophospholipid metabolism, fatty acid metabolism, and amino acid biosynthesis (Figure 4D–I). These results suggested that TG-driven divergence predominated at early and middle stages, particularly in XBJ, whereas later differences between HXJ and XBJ were mainly driven by lipid and amino acid metabolism rather than TGs alone. All these metabolomic data revealed distinct breed-specific metabolic profiles, with TGs emerging as central metabolites connecting growth patterns to meat quality, prompting subsequent transcriptomic analyses to identify the underlying regulatory mechanisms regulating these metabolic differences.

### 3.3. Transcriptomic Dynamics Reveal Upstream Regulators Underlying the Metabolic Divergence Driving the Growth-Quality Trade-Off

To explore the transcriptional mechanisms shaping the metabolic divergence among the three breeds, RNA-Seq analysis of pectoralis major across three developmental stages was conducted. A total of 25,045 genes were detected, of which low-abundance transcripts were removed before downstream analyses.

**Figure 5:**
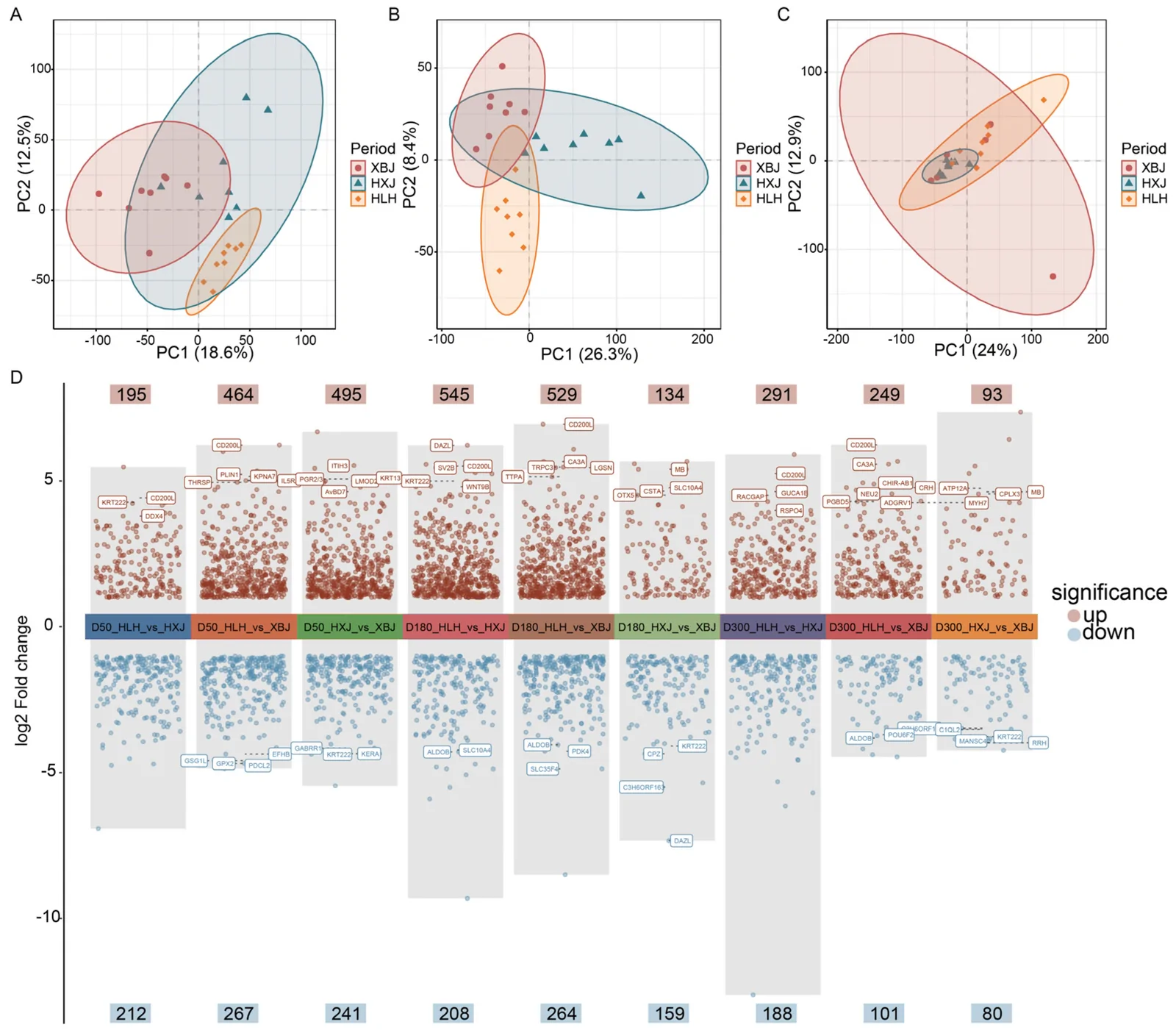
Differential gene expression in pectoralis major across three chicken breeds. (**A**–**C**) PCA scores of genes identified in three chicken breeds at 50 (**A**), 180 (**B**), and 300 (**C**) days of age. (**D**) Volcano plots showing differentially expressed genes among three breeds at the three developmental stages. *n* = 8 for omics analyses.

**Figure 6:**
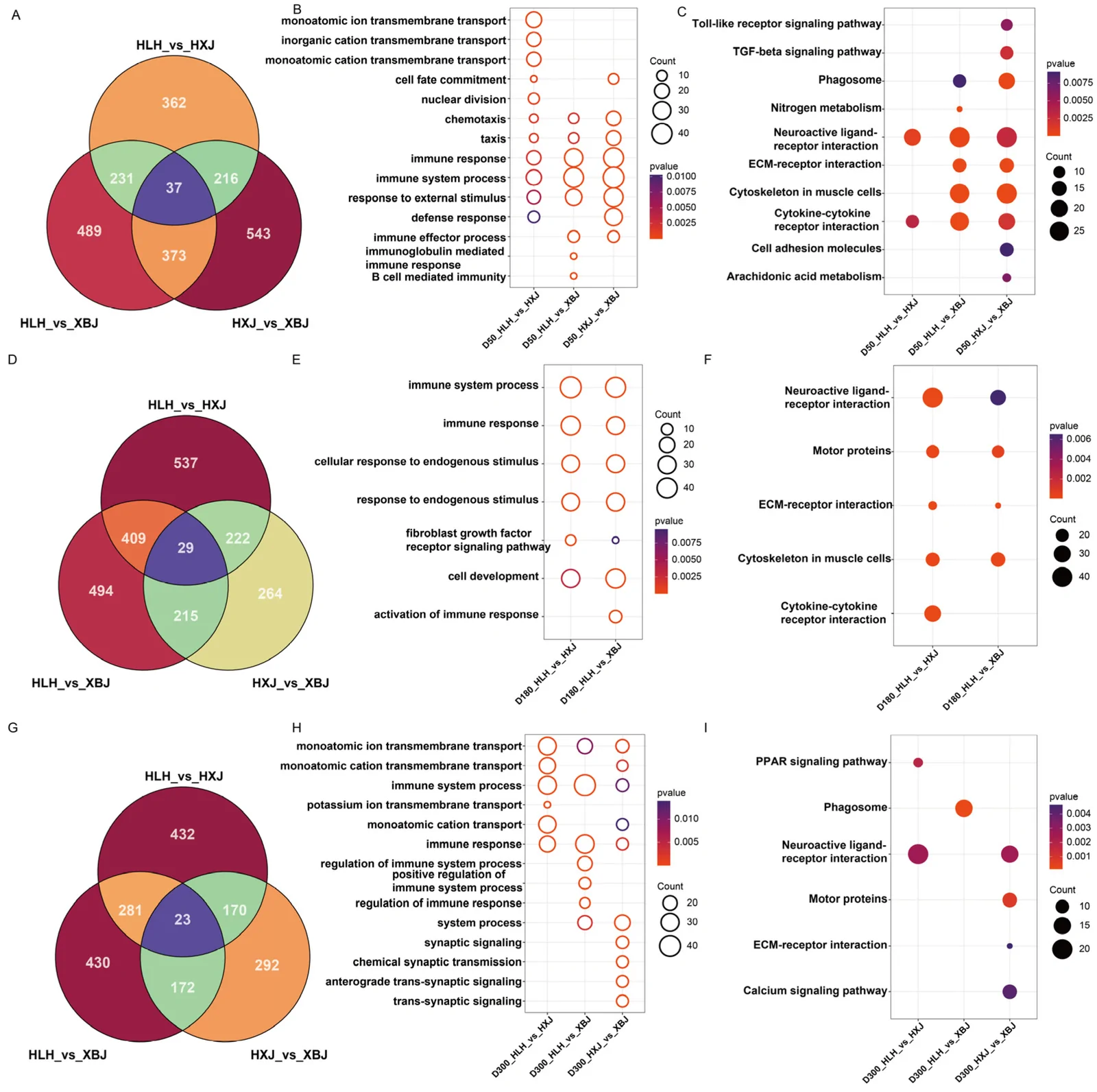
Enrichment analysis of differentially expressed genes in breast muscle across three chicken breeds. (**A**) Venn diagram of DEG numbers between XBJ, HXJ and HLH at 50 days of age. (**B**,**C**) Bubble plots showing GO (**B**) and KEGG (**C**) enrichment analysis of DEGs in pectoralis major of different chicken breeds at 50 days of age. (**D**) Venn diagrams depicting DEG counts across chicken breeds at 180 days of age. (**E**,**F**) GO (**E**) and KEGG (**F**) enrichment analysis bubble plots for pectoralis major DEGs in different chicken breeds at 180 days of age. (**G**) Venn diagrams representing DEG numbers among chicken breeds at 300 days of age. (**H**,**I**) Bubble plots of GO and KEGG enrichment analysis for pectoralis major DEGs in different chicken breeds at 300 days of age. *n* = 8 for omics analyses.

PCA revealed pronounced inter-breed transcriptomic divergence at 50 and 180 days (Figure 5A,B), whereas the expression profiles of XBJ, HXJ, and HLH at 300 days clustered together, with substantial overlap among the three breeds (Figure 5C). These temporal patterns indicated that breed-specific transcriptional divergence was largely established during early and middle development, whereas later stages were characterized by transcriptomic convergence. Consistent with the PCA results, the largest number of DEGs was detected at early and middle developmental stages, with a gradual reduction by 300 days (Figure 6). At 50 days, a total of 2251 DEGs were identified, including 1130 in XBJ vs. HLH, 1169 in XBJ vs. HXJ, and 846 in HXJ vs. HLH, with only 37 genes shared across all pairwise comparisons (Figure 6A). Furthermore, XBJ exhibited a distinct expression bias characterized by a greater number of upregulated genes (Figure 5D). At 180 days, a total of 2170 DEGs were identified, including 1147 in XBJ vs. HLH, 730 in XBJ vs. HXJ, and 1197 in HXJ vs. HLH, with 29 shared DEGs (Figure 6D). By 300 days, the number of DEGs decreased to 1800, including 906 in both XBJ vs. HLH and HXJ vs. HLH, and 657 in XBJ vs. HXJ, with 23 shared DEGs (Figure 6G). These results suggested that breed-specific transcriptional divergence was most pronounced at 50 and 180 days, consistent with the periods of greatest phenotypic and metabolic separation, whereas the reduced number of DEGs at 300 days reflected a relative convergence of gene expression profiles with age.

GO, KEGG, and Gene Set Enrichment Analysis (GSEA) were performed to explore the biological functions of these DEGs. Functional enrichment analyses revealed that breed-specific DEGs were enriched in pathways directly relevant to muscle growth, metabolism, and structural organization. At D50, DEGs distinguishing XBJ from the other two breeds, HXJ and HLH, were enriched in the cytoskeleton in muscle cells, cytokine–cytokine receptor interactions, ECM–receptor interaction (Figure 6 and Figure S3B for HLH vs. XBJ), TGF-β signaling pathway, cytoskeletal muscle cells, and ECM–receptor interaction (Figure 6 and Figure S3C for HXJ vs. XBJ), which were consistent with its rapid muscle fiber proliferation and remodeling. In contrast, DEGs between HXJ and HLH were primarily associated with fatty acid response, steroid biosynthesis, and motor proteins (Figure 6 and Figure S3A), reflecting their notable differences in energy metabolism balance and meat quality attributes at the early growth stage. By D180, both XBJ and HXJ continued to show enrichment in structural pathways when compared to HLH. Genes upregulated in XBJ were primarily enriched in ECM-receptor interaction and cytoskeletal in muscle cells, while HXJ exhibited elevated expression involved in ECM–receptor interaction, cytoskeletal in muscle cells, motor proteins, and neuroactive ligand–receptor interaction (Figure 6 and Figure S4). Furthermore, relative to HXJ, XBJ displayed additional upregulation of arginine and proline metabolism, TCA cycle activity, and ATP-dependent chromatin remodeling, and downregulated expression involved in motor proteins and neuroactive ligand–receptor interactions (Figure S4). These pathways were broadly associated with enhanced amino acid turnover, energy production, and transcriptional regulation, which were consistent with XBJ’s accelerated growth phenotype. At D300, the number of DEGs was further reduced across all pairwise comparisons, with the most notable differences observed between HXJ and HLH (Figure 6G). These DEGs were enriched in the PPAR signaling pathway, cell adhesion molecules, ECM-receptor interaction, gap junction, and motor protein-related pathways (Figure 6 and Figure S5). Compared to HLH, XBJ showed enhanced activity in phagosome, ECM-receptor interaction, focal adhesion, gap junction, and motor protein-related pathways, while genes related to the Polycomb repressive complex were downregulated (Figure S5). In the XBJ vs. HXJ comparison, DEGs were enriched in motor proteins and metabolic pathways, including oxidative phosphorylation, arachidonic acid metabolism, glycine, serine, and threonine metabolism, porphyrin metabolism, and pyrimidine metabolism with upregulated expression, while DEGs enriched in steroid hormone biosynthesis were downregulated (Figure 6 and Figure S5).

### 3.4. Integrated Analysis Identifies Key Genes and Metabolites Associated with Meat Quality

Correlation analyses between all differentially expressed metabolites (DEMs) and phenotypic traits were performed to explore potential associations between DEMs and meat quality traits. Metabolites with |r| > 0.7 and *p* < 0.05 were considered significantly associated and were retained for further analysis. Correlation analyses revealed distinct associations between DEMs and phenotypic traits across breeds and developmental stages (Table 2 and Figure S6). At D50, a total of 57 DEMs from HXJ and HLH were significantly correlated with breast muscle weight, a*value, and shear force (Table 2 and Figure S6), with all a*value-associated metabolites identified as triglycerides (TGs), including TG(14:0_18:1_20:2), TG(18:1_20:1_20:1), and TG(16:0_16:1_22:6). In the XBJ vs. HLH comparison, numerous DEMs were correlated with breast muscle weight, a* and b* values, and notably, 541 DEMs showed significant association with drip loss (Table 2 and Figure S6B), most of which were triglycerides (TGs) and phosphatidylethanolamines (PEs), suggesting that early-stage lipid metabolic modulation might be a major determinant of water-holding capacity. Similarly, a large number of DEMs between XBJ and HXJ were found to be associated with meat quality traits including drip loss, shear force, and a* value (Table 2 and Figure S6C). By D180 and D300, the number of significantly correlated metabolites declined sharply across all comparisons compared to D50, indicating that metabolic–phenotypic associations weakened as birds reached physiological maturity (Table 2 and Figure S6D–I). At D180, trait-associated metabolites showed increasing breed specificity. In HXJ vs. HLH, 29 DEMs were associated with breast muscle weight and 1 metabolite linked to pH (Table 2 and Figure S6D). In the comparison between XBJ and HLH, a total of 98 DEMs, primarily TGs and PEs, showed significant correlations with breast muscle weight, shear force, and L* value, highlighting continued TG-dominant contributions to meat color and texture (Table 2 and Figure S6E). In contrast, only 17 TGs or PEs were found to be correlated with breast muscle weight, and 2 with L* value in the XBJ vs. HXJ comparison, suggesting a reduced extent of metabolic divergence between XBJ and HXJ (Table 2 and Figure S6F). At D300, 10 DEMs were identified as being associated with shear force, and 3 with skin color in HXJ vs. HLH (Table 2 and Figure S6G). In XBJ vs. HLH, 141 DEMs were identified to be associated with meat quality traits such as shear force and drip loss in addition to breast muscle weight (Table 2 and Figure S6H), whereas 26 DEMs in XBJ vs. HXJ were found to be associated with breast muscle weight and drip loss (Table 2 and Figure S6I). These results suggested that while TGs and related lipids remained functionally relevant across all stages, their regulatory impact on meat quality traits diminished with maturation.

Similarly, correlations between all DEGs and phenotypic traits were analyzed. Based on the same criteria, breed- and age-specific DEGs were identified as being significantly associated with traits such as breast muscle weight, shear force, drip loss, and skin color (Table 3 and Figure S7). In the HXJ vs. HLH comparison, DEGs at D50 were primarily associated with shear force, breast muscle weight, and a value*. At D180, most DEGs were associated with shear force, while at D300, only a few remained correlated with shear force and L value*. For the XBJ vs. HLH group, DEGs at D50 were extensively associated with breast muscle weight, drip loss, and a value*, whereas at D180, the number of correlated DEGs markedly declined and the correlations shifted mainly toward breast muscle weight, shear force, L* value, and a* value. At D300, DEGs were mostly related to breast muscle weight, shear force, and drip loss (Table 3 and Figure S7). In XBJ vs. HXJ, numerous DEGs showed significant correlations with breast muscle weight, shear force, drip loss, and color parameters at D50. However, the number of significantly correlated genes decreased sharply at later stages. At D180, DEGs were mainly associated with breast muscle weight and L* value, while correlations were restricted to breast muscle weight and drip loss at D300 (Table 3 and Figure S7).

**Figure 7:**
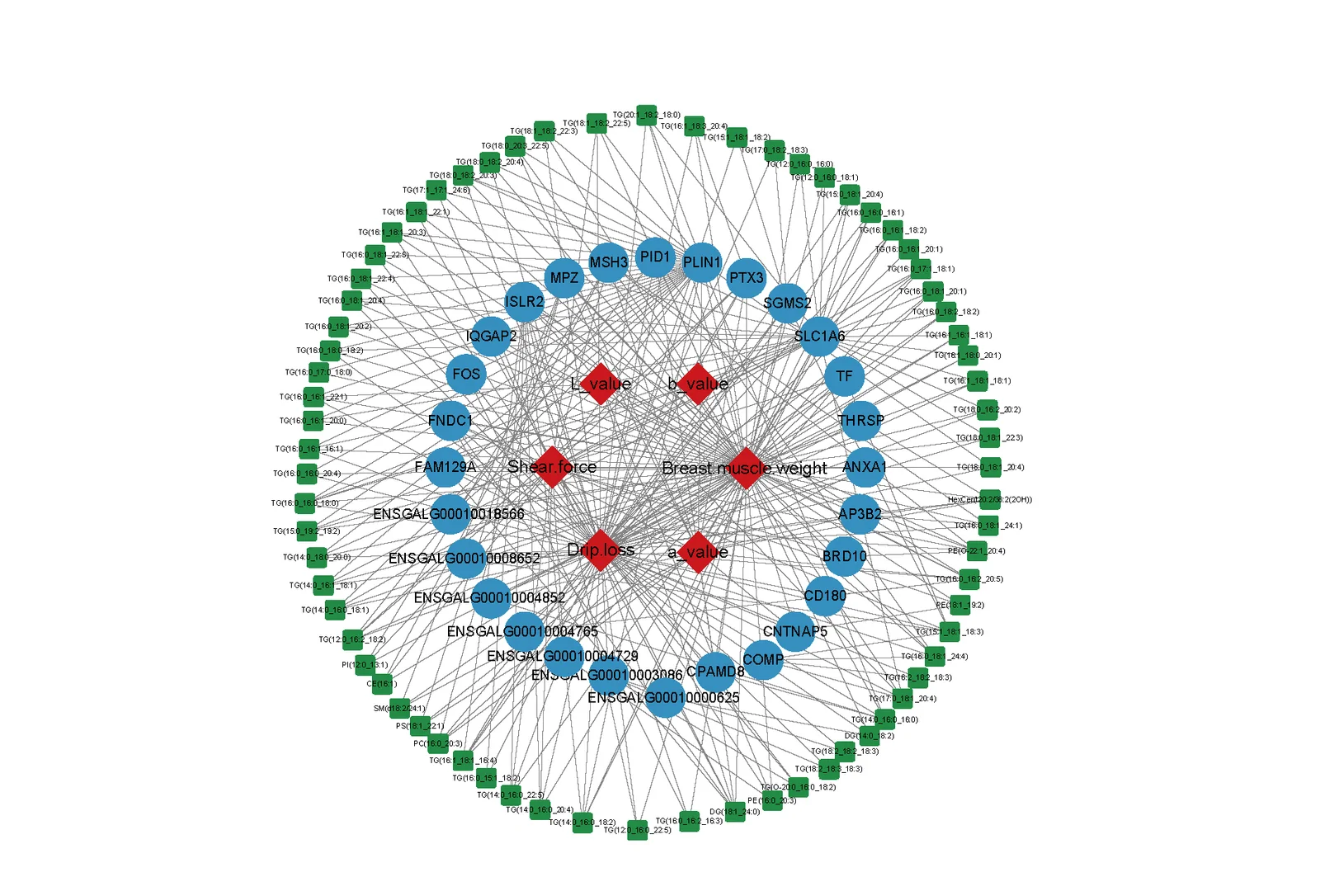
Association interaction network linking meat quality traits with genes and metabolites. Red diamonds denote meat quality traits, blue circles represent genes, and green rectangles indicate metabolites. *n* = 8 for omics analyses.

Finally, by integrating DEMs and DEGs associated with meat quality traits, 28 key regulatory genes and 71 key metabolites were identified. A comprehensive gene–metabolite–phenotype regulatory network was constructed, elucidating the molecular mechanisms underlying meat quality divergence among the three chicken breeds (Figure 7 and Table S1). Notably, among the DEMs linked to meat quality, triglycerides (TGs) were the most prominent class. Importantly, PLIN1 and SLC1A6 emerged as key regulators, each controlling a large number of triglyceride species, and may serve as pivotal genes influencing meat quality traits among XBJ, HXJ, and HLH. Moreover, the correlation network demonstrated that lipid metabolism-related genes such as PLIN1, THRSP, and SGMS2 and triglyceride metabolites were strongly connected with core phenotypic traits such as breast muscle weight, drip loss, and shear force (Figure 7 and Table S1). These findings suggested that lipid metabolic regulation played a central role in determining breed-specific differences in meat quality, potentially regulating both water-holding capacity and muscle texture.

## 4. Discussion

This study systematically investigated the metabolomic and transcriptomic dynamics of the pectoralis major in three different breeds, including XBJ, HXJ, and HLH, across different developmental stages (50, 180, and 300 days of age). Integration of molecular profiles and phenotypic meat quality traits established a comprehensive framework revealing how breed- and age-dependent differences in growth pattern, metabolism, and gene regulation collectively determine meat quality characteristics.

XBJ, a superior white-feathered hybrid derived from white-feathered boliers, yellow-feathered broilers, and layer chickens, exhibits rapid growth and high meat yield [17]. HXJ, a native breed from southern China, is recognized for its desirable sensory attributes [31], whereas HLH, traditionally valued for its reproductive performance, has received limited attention with regard to meat quality traits [16,32].

The three breeds investigated here represent distinct poultry genetic resources selected for divergent production objectives. While XBJ and HXJ are of particular regional importance in China, the inclusion of HLH provides a valuable outgroup representing intensive selection for reproductive rather than muscular traits. The comparison therefore offers insights that extend beyond any single market.

These findings confirmed breed-specific differences in both meat quality and muscle composition. XBJ consistently exhibited greater body weight at all time points, reflecting enhanced growth potential and muscle development. However, at 50 days of age, HXJ and HLH exhibited redder breast muscle color and lower drip loss compared to XBJ. By 180 and 300 days, HLH showed significantly higher shear force values than XBJ, while pH values remained comparable among XBJ, HXJ, and HLH. Consistent with previous reports, chickens with larger body size at early stages tend to exhibit redder skin, although this trend may reverse as the birds age [33]. Notably, XBJ exhibited higher shear force than HXJ and HLH at 50 days, whereas HLH surpassed both XBJ and HXJ at later stages. This difference reflects variation in growth patterns: XBJ reaches market weight by 50 days, while HXJ and HLH, which have slower growth dynamics, continue muscle deposition over a longer period. Previous studies have similarly demonstrated that growth rate plays a critical role in determining meat quality traits [23,34]. Moreover, phenotypic analysis revealed a distinct trade-off between growth efficiency and meat quality among the three breeds, in which selection for rapid growth in XBJ was accompanied by reduced meat quality, whereas HXJ maintained superior quality at the expense of growth efficiency. On the other hand, HLH, representing a dual-purpose type, exhibited the toughest texture and lowest water retention, probably related to muscle fiber atrophy and excessive collagen accumulation associated with its egg-laying physiology [16]. These contrasting features reflected breed-specific developmental and metabolic adaptations that resulted in divergent growth rates and meat quality characteristics.

Metabolites in meat play a critical role in meat quality and flavor development [23,35]. Previous studies have identified that meat quality and metabolite profiles differ greatly between fast- and slow-growing chickens [36]. In the present study, metabolic profiles of different chicken breeds across different developmental stages were systematically compared. Metabolomic profiling revealed that lipid metabolism played a central role in regulating breed-specific meat quality. At D50, XBJ showed a marked enrichment of TGs compared with HXJ and HLH, indicating enhanced lipid deposition and energy mobilization to sustain rapid muscle growth. HXJ presented intermediate TG abundance and relatively higher levels of GPs, which are essential for membrane structure and oxidative stability. Previous studies have shown that GPs participate in multiple physiological processes such as oxidative phosphorylation and signal transduction, suggesting their active role in cellular energy metabolism [25,37,38,39]. In the pectoral muscle, GPs have been demonstrated to be involved not only in muscle cell metabolism and oxidative phosphorylation but also to serve as lipid reserves for egg formation in laying hens [38]. In this study, HLH was found to exhibit lower TG content but relatively higher proportions of GPs, indicating a shift toward oxidative energy metabolism and membrane maintenance in HLH, which was consistent with its physiological adaptation for prolonged egg production rather than rapid muscle growth. As birds matured, TG abundance declined among XBJ, HXJ, and HLH, and metabolic profiles shifted toward lipid and amino acid metabolism, suggesting that energy-demanding lipid accumulation during early growth transitioned into metabolic maintenance and structural preservation in later stages. A similar age-related reduction in differential metabolites has been reported in Beijing-You chickens, supporting the notion that metabolic activity becomes more stable as birds reach later developmental stages [14]. Meanwhile, these observations were consistent with previous research showing that amino acid and lipid metabolism are major contributors to the differences in meat quality and flavor between indigenous breeds and commercial chickens [23,36,40].

Transcriptomic analysis supported these metabolic distinctions. The three breeds exhibited distinct transcriptional profiles from the early growth stage, with the most pronounced divergence observed at 50 and 180 days. DEG analysis showed that XBJ consistently exhibited a higher number of upregulated genes across multiple time points, suggesting enhanced muscle development and elevated metabolic activity. Genes upregulated in XBJ were associated with ECM-receptor interaction, cytoskeletal organization, and TGF-β signaling, consistent with rapid muscle fiber proliferation and structural reorganization in the pectoralis major. HXJ showed enrichment in fatty acid response, steroid biosynthesis, and motor protein pathways, reflecting precise regulation of lipid utilization and maintenance of muscle structural and contractile functions. In contrast, genes expressed in HLH were found to be enriched in oxidative metabolism, gap junctions, and collagen organization, consistent with its endurance-type muscle physiology. As the chickens aged, gene expression differences narrowed, suggesting that transcriptional profiles stabilized and became more similar across breeds at later stages. Similar age-dependent transcriptomic and metabolite profiles of meat quality have also been reported in Jingyuan chickens [25], suggesting that developmental timing is a primary determinant of molecular differentiation among breeds. Furthermore, in this study, lipid metabolism emerged as a central axis connecting transcriptional regulation with phenotypic traits. It played a major role in coordinating muscle structure, energy use, and meat texture among XBJ, HXJ, and HLH. XBJ depended on lipid-driven energy deposition for rapid growth, and HXJ exhibited a more balanced metabolic profile associated with improved tenderness and color, whereas HLH showed slower lipid turnover and higher glycerophospholipid proportions related to membrane stability. These differences illustrated how lipid metabolism helped balance growth efficiency and meat quality, with metabolic, structural, and transcriptional regulation jointly determining the observed phenotypic diversity in meat quality across chicken breeds.

Integrative analysis of transcriptomics and metabolomics is an effective approach to uncover the molecular basis of chicken meat quality [41]. In this study, integrative analysis of differential metabolites and genes provided deeper insight into the molecular mechanisms connecting lipid metabolism with meat quality traits. This analysis led to the construction of a regulatory interaction network comprising 28 key genes and 71 key metabolites. Correlation networks revealed that TGs and phosphatidylethanolamines (PEs) were the main metabolite classes associated with drip loss, shear force, and muscle weight, particularly at early stages. The number of significant metabolite–trait correlations decreased markedly with age, indicating that metabolic control of meat quality weakened as the birds reached maturity. Among the identified genes, PLIN1 and SLC1A6 exhibited the most extensive regulatory influence over TG species. PLIN1 showed a strong negative correlation with drip loss (r = −0.77), highlighting its potential role in water-holding capacity regulation. Perilipins (PLINs) are structural proteins that play a critical role in intracellular lipid storage [42]. PLIN1, encoded by the PLIN1 gene, localizes to the lipid droplet membrane [43]. In goose breast muscle, phosphorylation of PLIN1 has been suggested to positively affect intramuscular fat (IMF) deposition [44], and in pigs it has been proposed as a candidate gene for growth, carcass, and meat quality [42]. Moreover, PLIN1 overexpression in glioma cells enhances lipid accumulation by upregulating genes involved in lipid biosynthesis and downregulating those involved in lipolysis [45]. Consistent with a central role for lipid droplet regulation in avian muscle, a transcriptomic study in Jingxing Yellow chickens reported that high-triglyceride birds showed coordinated changes in adipogenesis- and lipid-handling genes, including PLIN1, FABP4, FABP5, and PPARG, alongside five steroid biosynthesis genes [46]. This pattern supported a network in which PLIN1 operated together with other genes, thereby facilitating triglyceride accumulation within muscle. In this study, strong correlations between PLIN1 and multiple TG species further implicated PLIN1 as a key node connecting lipid droplet biology to meat quality-related lipid deposition, whereby elevated PLIN1 expression may enhance lipid retention and improve water-holding capacity. SLC1A6, a sodium-dependent glutamate transporter, is primarily involved in neuronal function, neuroprotection, and glutamate homeostasis [47,48]. Previous studies have mainly focused on its role in disease contexts [49,50,51], with little research on its relationship to TG metabolism. Interestingly, this study found that SLC1A6 exhibited extensive correlations with TG species, implying an indirect role in energy balance and lipid homeostasis. These genes appeared to act as key regulatory nodes connecting transcriptional regulation, metabolism, and meat quality traits.

Several limitations should be acknowledged. First, the initial data digitization inadvertently omitted individual pen identities, which constrained the first round of statistical analysis to one-way ANOVA. Upon re-examining the original sampling records, we retrieved the pen information and re-analyzed all phenotypic traits using linear mixed-effects models with pen nested within breed as a random effect. Although pen variance components were generally small, future studies should ensure that pen identities are accurately recorded from the outset to facilitate more robust mixed-effects modeling. Second, the standardized environment and chronological age feeding phases enabled direct genetic comparisons but may not reflect optimal commercial conditions for each breed. Third, the metabolomic and transcriptomic analyses were performed on eight birds per group, and the causal relationships between PLIN1/SLC1A6 and meat quality remain correlative; future studies with larger sample sizes and functional validation are needed. Finally, the selected breeds are commercially relevant in China, and only female chickens were studied; therefore, the results should be interpreted within this specific context, and sex-specific effects warrant further investigation.

## 5. Conclusions

In summary, this study systematically compared the meat quality, transcriptomic, and metabolomic profiles of three genetically distinct chicken breeds across developmental stages. Significant breed- and age-related differences were observed in body weight, meat color, drip loss, and shear force, reflecting diverse growth and metabolic profiles among fast-growing, slow-growing, and layer-type chickens. Integrative transcriptomic-metabolomic analyses revealed that lipid metabolism played a central role in regulating meat quality, with triglycerides and glycerophospholipids identified as the predominant differential metabolites. The genes *PLIN1* and *SLC1A6* were identified as key regulators of multiple triglyceride species, potentially influencing water-holding capacity and tenderness. These findings provide a comprehensive molecular framework connecting lipid metabolism to breed- and age-dependent meat quality formation. The outcomes deepen the understanding of the biological basis of chicken meat quality and offer potential molecular targets for improving meat quality in poultry breeding.

## Figures and Tables

**Table 1:** Analyzed nutrient levels of the experimental diets (as-fed basis).

Item	Starter (0–30 Days)	Grower (31–60 Days)	Finisher (61–300 Days)
Moisture (%)	9.6	10.6	10.7
Crude protein (%)	21.30	17.42	15.97
Crude fat (%)	4.2	3.3	3.9
Crude ash (%)	6.6	7.2	7.3
Crude fiber (%)	3.4	4.2	4.6
Total phosphorus (%)	0.68	0.81	0.64
Water-soluble chloride (%)	0.3	0.3	0.4
Metabolizable energy (MJ/kg)	12.62	11.86	11.78

Note: Values for moisture, crude protein, crude fat, crude ash, crude fiber, total phosphorus, and water-soluble chloride were determined by standard laboratory methods using representative feed samples collected at each feeding phase. Metabolizable energy (ME) was calculated from the proximate composition according to the following equation: ME (kcal/kg) = 40.81 × CP (%) + 81.27 × EE (%) + 35.45 × NFE (%) − 41.68 × CF (%), where NFE (%) = 100 − moisture (%) − CP (%) − EE (%) − ash (%) − CF (%); values were then converted to MJ/kg (1 kcal = 4.184 kJ).

**Table 2:** Top five differential metabolites correlated with meat quality traits in each comparison group.

Group	Metabolite	Phenotype	Correlation	*p*_Value
D50 HXJ vs. HLH	TG(14:0_16:1_20:4)	Breast.muscle.weight	0.88	7.65 × 10^−6^
D50 HXJ vs. HLH	TG(16:0_18:1_24:1)	Breast.muscle.weight	0.85	3.38 × 10^−5^
D50 HXJ vs. HLH	TG(18:1_20:1_20:1)	Breast.muscle.weight	0.83	5.95 × 10^−5^
D50 HXJ vs. HLH	TG(14:0_18:1_20:2)	Breast.muscle.weight	0.81	1.47 × 10^−4^
D50 HXJ vs. HLH	HexCer(t20:2/38:2(2OH))	Breast.muscle.weight	0.80	1.89 × 10^−4^
D50 XBJ vs. HLH	SM(d18:2/24:1)	Breast.muscle.weight	−0.97	1.18 × 10^−9^
D50 XBJ vs. HLH	PC(16:0_20:3)	Breast.muscle.weight	−0.96	3.58 × 10^−9^
D50 XBJ vs. HLH	PE(O-20:3_20:4)	Breast.muscle.weight	−0.96	8.58 × 10^−9^
D50 XBJ vs. HLH	PE(20:2_18:0)	Breast.muscle.weight	0.95	1.02 × 10^−8^
D50 XBJ vs. HLH	PA(18:1_26:1)	Breast.muscle.weight	−0.94	9.44 × 10^−8^
D50 HXJ vs. XBJ	Orotic Acid	Breast.muscle.weight	0.98	1.84 × 10^−11^
D50 HXJ vs. XBJ	PC(16:0_20:3)	Breast.muscle.weight	−0.95	1.10 × 10^−8^
D50 HXJ vs. XBJ	HexCer(d18:1/22:0)	Breast.muscle.weight	−0.94	4.04 × 10^−8^
D50 HXJ vs. XBJ	2-Hydroxybutanoic Acid	Breast.muscle.weight	−0.93	2.29 × 10^−7^
D50 HXJ vs. XBJ	3-Hydroxybutanoic acid	Breast.muscle.weight	−0.93	2.29 × 10^−7^
D180 HXJ vs. HLH	LPE(22:3/0:0)	Shear.force	−0.87	1.09 × 10^−5^
D180 HXJ vs. HLH	Guanidine	Shear.force	−0.85	3.16 × 10^−5^
D180 HXJ vs. HLH	PI(12:0_13:1)	Shear.force	−0.85	3.02 × 10^−5^
D180 HXJ vs. HLH	Asn-Asn	Shear.force	0.83	8.04 × 10^−5^
D180 HXJ vs. HLH	PE(18:0_22:1)	Shear.force	−0.83	7.76 × 10^−5^
D180 XBJ vs. HLH	PE(O-22:1_20:4)	Breast.muscle.weight	0.99	4.11 × 10^−12^
D180 XBJ vs. HLH	PE(20:2_18:0)	Breast.muscle.weight	0.96	8.09 × 10^−9^
D180 XBJ vs. HLH	PI(12:0_13:1)	Breast.muscle.weight	0.95	9.24 × 10^−9^
D180 XBJ vs. HLH	TG(16:0_16:0_16:0)	Breast.muscle.weight	0.94	8.85 × 10^−8^
D180 XBJ vs. HLH	FFA(22:2)	Breast.muscle.weight	0.93	2.23 × 10^−7^
D180 HXJ vs. XBJ	PE(O-22:1_20:4)	Breast.muscle.weight	0.98	5.87 × 10^−12^
D180 HXJ vs. XBJ	PE(16:0_20:3)	Breast.muscle.weight	−0.92	3.06 × 10^−7^
D180 HXJ vs. XBJ	TG(16:0_16:0_18:0)	Breast.muscle.weight	0.88	5.74 × 10^−6^
D180 HXJ vs. XBJ	TG(18:0_18:2_20:2)	Breast.muscle.weight	0.82	1.06 × 10^−4^
D180 HXJ vs. XBJ	TG(18:0_18:1_20:3)	Breast.muscle.weight	0.8	1.88 × 10^−4^
D300 HXJ vs. HLH	PS(20:0_16:1)	Shear.force	−0.84	3.85 × 10^−5^
D300 HXJ vs. HLH	PE(22:1_18:0)	Shear.force	−0.82	1.18 × 10^−4^
D300 HXJ vs. HLH	PE(18:0_22:1)	Shear.force	−0.77	5.21 × 10^−4^
D300 HXJ vs. HLH	Guanidine	Shear.force	0.74	1.00 × 10^−3^
D300 HXJ vs. HLH	TG(16:0_16:0_16:0)	b* value	0.73	1.30 × 10^−3^
D300 XBJ vs. HLH	PS(20:0_16:1)	Breast.muscle.weight	0.96	2.04 × 10^−9^
D300 XBJ vs. HLH	CE(16:1)	Breast.muscle.weight	−0.96	7.67 × 10^−9^
D300 XBJ vs. HLH	PS(18:2_16:0)	Breast.muscle.weight	−0.94	1.09 × 10^−7^
D300 XBJ vs. HLH	PE(P-18:1_20:0)	Breast.muscle.weight	−0.93	1.15 × 10^−7^
D300 XBJ vs. HLH	PE(O-16:0_20:0)	Breast.muscle.weight	−0.92	5.17 × 10^−7^
D300 HXJ vs. XBJ	Guanidine	Breast.muscle.weight	0.98	5.73 × 10^−11^
D300 HXJ vs. XBJ	DG(15:1_16:1)	Breast.muscle.weight	0.96	3.49 × 10^−9^
D300 HXJ vs. XBJ	Methyldopa	Breast.muscle.weight	0.93	2.88 × 10^−7^
D300 HXJ vs. XBJ	Guanidinoethyl Sulfonate	Breast.muscle.weight	−0.93	1.65 × 10^−7^
D300 HXJ vs. XBJ	Orotic Acid	Breast.muscle.weight	0.9	2.53 × 10^−6^

**Table 3:** Top five differentially expressed genes correlated with meat quality traits in each comparison group.

Group	Gene	Phenotype	Correlation	*p*_Value
D50 HXJ vs. HLH	ENSGALG00010019171	Shear.force	−0.91	9.46 × 10^−7^
D50 HXJ vs. HLH	ENSGALG00010020240	Breast.muscle.weight	0.88	8.66 × 10^−6^
D50 HXJ vs. HLH	ENSGALG00010025979	a* value	−0.87	1.44 × 10^−5^
D50 HXJ vs. HLH	ENSGALG00010000662	Shear.force	−0.87	1.32 × 10^−5^
D50 HXJ vs. HLH	ENSGALG00010017970	Breast.muscle.weight	0.87	1.52 × 10^−5^
D50 XBJ vs. HLH	ENSGALG00010027487	Breast.muscle.weight	−0.98	1.05 × 10^−11^
D50 XBJ vs. HLH	ENSGALG00010027487	a* value	0.85	3.30 × 10^−5^
D50 XBJ vs. HLH	ENSGALG00010016053	Breast.muscle.weight	−0.93	2.81 × 10^−7^
D50 XBJ vs. HLH	ENSGALG00010008182	Breast.muscle.weight	0.92	3.22 × 10^−7^
D50 XBJ vs. HLH	ENSGALG00010004993	a* value	−0.91	1.05 × 10^−6^
D50 HXJ vs. XBJ	ENSGALG00010008126	Breast.muscle.weight	0.94	9.61 × 10^−8^
D50 HXJ vs. XBJ	ENSGALG00010010896	Breast.muscle.weight	0.93	2.42 × 10^−7^
D50 HXJ vs. XBJ	ENSGALG00010027487	Breast.muscle.weight	−0.93	1.42 × 10^−7^
D50 HXJ vs. XBJ	ENSGALG00010025982	a* value	−0.92	5.58 × 10^−7^
D50 HXJ vs. XBJ	ENSGALG00010014018	a* value	−0.92	3.07 × 10^−7^
D180 HXJ vs. HLH	ENSGALG00010027636	Shear.force	−0.91	1.30 × 10^−6^
D180 HXJ vs. HLH	ENSGALG00010003724	Shear.force	0.89	4.73 × 10^−6^
D180 HXJ vs. HLH	ENSGALG00010007217	Shear.force	0.88	7.21 × 10^−6^
D180 HXJ vs. HLH	ENSGALG00010000651	Shear.force	−0.88	8.80 × 10^−6^
D180 HXJ vs. HLH	ENSGALG00010017887	Shear.force	−0.87	1.43 × 10^−5^
D180 XBJ vs. HLH	ENSGALG00010007217	Breast.muscle.weight	−0.97	9.46 × 10^−10^
D180 XBJ vs. HLH	ENSGALG00010018566	Breast.muscle.weight	−0.92	4.68 × 10^−7^
D180 XBJ vs. HLH	ENSGALG00010028348	Breast.muscle.weight	0.91	7.16 × 10^−7^
D180 XBJ vs. HLH	ENSGALG00010022238	Breast.muscle.weight	0.9	1.86 × 10^−6^
D180 XBJ vs. HLH	ENSGALG00010026555	Breast.muscle.weight	0.9	1.73 × 10^−8^
D180 HXJ vs. XBJ	ENSGALG00010012952	Breast.muscle.weight	−0.95	2.74 × 10^−8^
D180 HXJ vs. XBJ	ENSGALG00010021505	b* value	−0.89	3.03 × 10^−6^
D180 HXJ vs. XBJ	ENSGALG00010015774	L* value	0.89	4.14 × 10^−6^
D180 HXJ vs. XBJ	ENSGALG00010003761	Breast.muscle.weight	−0.88	6.11 × 10^−6^
D180 HXJ vs. XBJ	ENSGALG00010004729	Breast.muscle.weight	−0.86	1.56 × 10^−5^
D300 HXJ vs. HLH	ENSGALG00010004707	Shear.force	−0.91	1.05 × 10^−6^
D300 HXJ vs. HLH	ENSGALG00010002481	Shear.force	−0.89	3.66 × 10^−6^
D300 HXJ vs. HLH	ENSGALG00010003528	Shear.force	−0.85	2.65 × 10^−5^
D300 HXJ vs. HLH	ENSGALG00010019781	L* value	0.85	2.52 × 10^−5^
D300 HXJ vs. HLH	ENSGALG00010000486	Shear.force	−0.84	4.04 × 10^−5^
D300 XBJ vs. HLH	ENSGALG00010011937	Breast.muscle.weight	−0.96	6.98 × 10^−9^
D300 XBJ vs. HLH	ENSGALG00010007217	Breast.muscle.weight	−0.96	2.22 × 10^−9^
D300 XBJ vs. HLH	ENSGALG00010020835	Breast.muscle.weight	0.94	5.21 × 10^−8^
D300 XBJ vs. HLH	ENSGALG00010016875	Breast.muscle.weight	−0.92	6.63 × 10^−7^
D300 XBJ vs. HLH	ENSGALG00010008429	Breast.muscle.weight	−0.92	4.31 × 10^−7^
D300 HXJ vs. XBJ	ENSGALG00010009400	Breast.muscle.weight	−0.89	4.10 × 10^−6^
D300 HXJ vs. XBJ	ENSGALG00010012952	Breast.muscle.weight	−0.87	9.20 × 10^−6^
D300 HXJ vs. XBJ	ENSGALG00010015131	Breast.muscle.weight	−0.86	2.21 × 10^−5^
D300 HXJ vs. XBJ	ENSGALG00010010929	Breast.muscle.weight	0.85	3.03 × 10^−5^
D300 HXJ vs. XBJ	ENSGALG00010001914	Drip.loss	0.84	5.40 × 10^−5^

## Data Availability

The original data presented in the study are available in the Genome Sequence Archive (GSA) at the China National Center for Bioinformation (CNCB) under accession number CRA042036.

## Supplementary Materials

The following supporting information can be downloaded at: https://www.mdpi.com/article/10.3390/genes17091036/s1, Figure S1: Model quality evaluation of OPLS-DA models. (A-C) Model quality assessment of OPLS-DA between HLH and HXJ (A), HLH and XBJ (B), and HXJ and XBJ (C) at D50. (D–F) Model quality assessment of OPLS-DA between HLH and HXJ (D), HLH and XBJ (E), and HXJ and XBJ (F) at D180. (G–I) Model quality assessment of OPLS-DA between HLH and HXJ (G), HLH and XBJ (H), and HXJ and XBJ (I) at D300; Figure S2: Classification of differentially expressed metabolites (DEMs). (A–C) Classification of DEMs between HLH and HXJ (A), HLH and XBJ (B), and HXJ and XBJ (C) at D50. (D–F) Classification of DEMs between HLH and HXJ (D), HLH and XBJ (E), and HXJ and XBJ (F) at D180. (G–I) Classification of DEMs between HLH and HXJ (G), HLH and XBJ (H), and HXJ and XBJ (I) at D300; Figure S3: GSEA of differentially expressed genes at D50. (A–C) GSEA-GO enrichment analysis of genes between HLH and HXJ (A), HLH and XBJ (B), and HXJ and XBJ (C). (D–F) GSEA-KEGG enrichment analysis of genes between HLH and HXJ (D), HLH and XBJ (E), and HXJ and XBJ (F); Figure S4: GSEA analysis of differentially expressed genes at D180. (A-C) GSEA-GO enrichment analysis of genes between HLH and HXJ (A), HLH and XBJ (B), and HXJ and XBJ (C). (D–F) GSEA-KEGG enrichment analysis of genes between HLH and HXJ (D), HLH and XBJ (E), and HXJ and XBJ (F); Figure S5: GSEA analysis of differentially expressed genes at D300. (A–C) GSEA-GO enrichment analysis of genes between HLH and HXJ (A), HLH and XBJ (B), and HXJ and XBJ (C). (D–F) GSEA-KEGG enrichment analysis of genes between HLH and HXJ (D), HLH and XBJ (E), and HXJ and XBJ (F). Figure S6: Correlation analysis between differential metabolites and phenotypes in different chicken breeds. (A–C) Correlation network diagrams between differential metabolites in the pectoralis major and meat quality traits of HXJ and HLH (A), XBJ and HLH (B), and HXJ and XBJ (C) at 50 days of age. (D–F) Correlation network diagrams between differential metabolites in the pectoralis major and meat quality traits of HXJ and HLH (D), XBJ and HLH (E), and HXJ and XBJ (F) at 180 days of age. (G–I) Correlation network diagrams between differential metabolites in the pectoralis major and meat quality traits of HXJ and HLH (G), XBJ and HLH (H), and HXJ and XBJ (I) at 300 days of age. Figure S7: Correlation analysis of differentially expressed genes and meat quality traits in distinct chicken breeds. (A–C) Correlation networks between DEGs and meat quality traits in pectoralis major of HXJ vs. HLH (A), XBJ vs. HLH (B), and HXJ vs. XBJ (C) at 50 days of age. (D–F) Correlation networks between DEGs and meat quality traits in pectoralis major of HXJ vs. HLH (D), XBJ vs. HLH (E), and HXJ vs. XBJ (F) at 180 days of age. (G–I) Correlation networks between DEGs and meat quality traits in pectoralis major of HXJ vs. HLH (G), XBJ vs. HLH (H), and HXJ vs. XBJ (I) at 300 days of age. Table S1: Correlation between key hub genes and metabolites.

## Abbreviations

The following abbreviations are used in this manuscript:

BMW	Breast muscle weight
ECM remodeling	Extracellular matrix remodeling
LC-MS	Liquid Chromatography-Mass Spectrometry
PCA	Principal component analysis
DEGs	Differentially expressed genes
DEMs	Differentially expressed metabolites
