# Supplementary material for: Integrated Multi-Omics Analysis Reveals Lipid Metabolism as a Key Contributor to the Growth–Meat Quality Trade-Off Among Genetically Divergent Chicken Breeds
Source: Genes (Basel). 2026 Aug 29;17(9):1036. doi: 10.3390/genes17091036 (PMC13606430; doi:10.3390/genes17091036)
Supplement: Supplementary file 1 [file genes-17-01036-s001.zip › Figure S6.pdf]

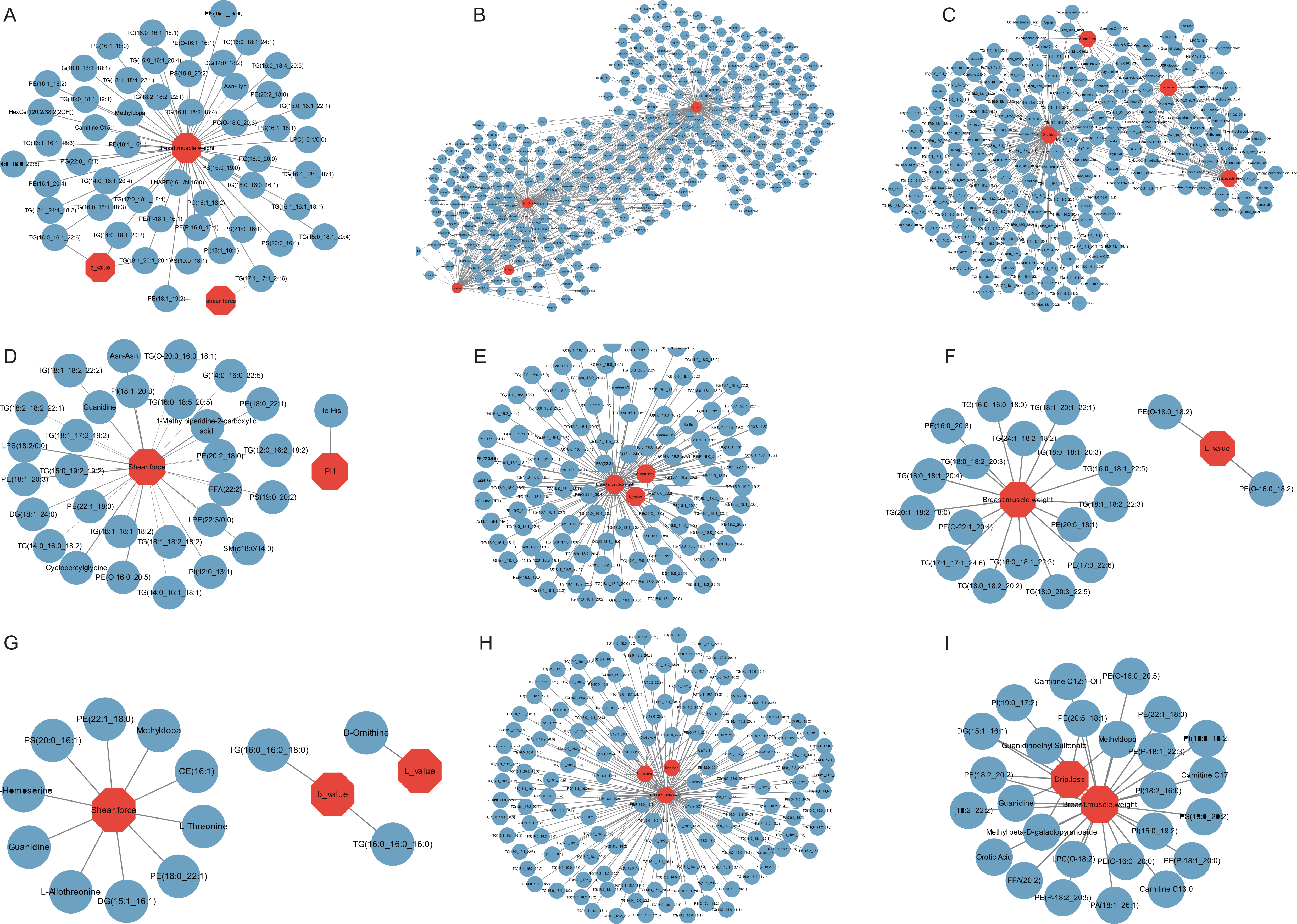
A
B
C
D
E
F
G
H
I
TG(16:0_16:1_16:1)
PE(16:1_18:0)
PE(O-16:1_16:1)
TG(16:0_18:1_24:1)
TG(16:0_16:1_20:4)
DG(14:0_18:2)
TG(16:0_18:4_20:5)
TG(16:0_18:1_18:1)
TG(18:1_18:1_22:1)
PS(19:0_20:2)
Asn-Hyp
PE(16:1_18:2)
PE(20:2_18:0)
TG(18:0_18:1_19:1)
TG(18:2_18:2_22:1)
TG(15:0_18:1_22:1)
Methyldopa
HexCer(t20:2/38:2(2OH))
TG(16:0_18:2_18:4)
Carnitine C15:1
PC(O-18:0_20:3)
PC(16:1_16:1)
TG(16:1_16:1_18:3)
PE(18:1_16:1)
Breast.muscle.weight
LPC(16:1/0:0)
PG(22:0_16:1)
PG(16:0_20:0)
PS(16:0_19:0)
TG(16:1_18:1_18:1)
PE(16:1_20:4)
TG(14:0_16:1_20:4)
LNAPE(16:1/N-16:0)
TG(16:0_16:0_16:1)
TG(17:0_18:1_18:1)
PC(16:1_18:2)
TG(18:1_24:1_18:2)
TG(16:0_16:1_18:3)
TG(16:1_16:1_18:1)
PE(P-18:1_16:1)
PE(P-16:0_16:1)
PS(21:0_16:1)
TG(16:0_16:1_22:6)
TG(14:0_18:1_20:2)
TG(15:0_18:1_20:4)
PS(20:0_16:1)
PI(18:1_18:1)
a_value
TG(18:1_20:1_20:1)
PS(19:0_18:1)
TG(17:1_17:1_24:6)
PE(18:1_19:2)
shear force
Asn-Asn
TG(O-20:0_16:0_18:1)
TG(18:1_18:2_22:2)
PI(18:1_20:3)
TG(14:0_16:0_22:5)
Guanidine
TG(16:0_18:5_20:5)
PE(18:0_22:1)
Ile-His
TG(18:2_18:2_22:1)
1-Methylpiperidine-2-carboxylic acid
TG(18:1_17:2_19:2)
LPS(18:2/0:0)
PE(20:2_18:0)
TG(12:0_16:2_18:2)
Shear.force
PH
TG(15:0_19:2_19:2)
PE(18:1_20:3)
FFA(22:2)
DG(18:1_24:0)
PE(22:1_18:0)
PS(19:0_20:2)
LPE(22:3/0:0)
TG(18:1_18:1_18:2)
TG(14:0_16:0_18:2)
TG(18:1_18:2_18:2)
SM(d18:0/14:0)
Cyclopentylglycine
PE(O-16:0_20:5)
PI(12:0_13:1)
TG(14:0_16:1_18:1)
TG(16:0_16:0_18:0)
TG(18:1_20:1_22:1)
PE(O-18:0_18:2)
PE(16:0_20:3)
TG(24:1_18:2_18:2)
L_value
TG(18:0_18:2_20:3)
TG(18:0_18:1_20:3)
TG(18:0_18:1_20:4)
TG(16:0_18:1_22:5)
PE(O-16:0_18:2)
TG(18:1_18:2_22:3)
TG(20:1_18:2_18:0)
PE(O-22:1_20:4)
PE(20:5_18:1)
TG(17:1_17:1_24:6)
TG(18:0_18:1_22:3)
PE(17:0_22:6)
TG(18:0_18:2_20:2)
TG(18:0_20:3_22:5)
PE(22:1_18:0)
PS(20:0_16:1)
Methyldopa
D-Ornithine
CE(16:1)
b_value
TG(16:0_16:0_16:0)
L-Threonine
Guanidine
PE(18:0_22:1)
L-Allothreonine
DG(15:1_16:1)
Carnitine C12:1-OH
PE(O-16:0_20:5)
PI(19:0_17:2)
PE(20:5_18:1)
PE(22:1_18:0)
DG(15:1_16:1)
Guanidinoethyl Sulfonate
PE(P-18:1_22:3)
PE(18:2_20:2)
Drip.loss
Carnitine C17
PI(18:2_16:0)
Breast.muscle.weight
Methyl beta-D-galactopyranoside
PI(15:0_19:2)
Orotic Acid
LPC(O-18:2)
PE(O-16:0_20:0)
PE(P-18:1_20:0)
FFA(20:2)
Carnitine C13:0
PE(P-18:2_20:5)
PA(18:1_26:1)
