# Supplementary material for: Integrated Multi-Omics Analysis Reveals Lipid Metabolism as a Key Contributor to the Growth–Meat Quality Trade-Off Among Genetically Divergent Chicken Breeds
Source: Genes (Basel). 2026 Aug 29;17(9):1036. doi: 10.3390/genes17091036 (PMC13606430; doi:10.3390/genes17091036)
Supplement: Supplementary file 1 [file genes-17-01036-s001.zip › Figure S7.pdf]

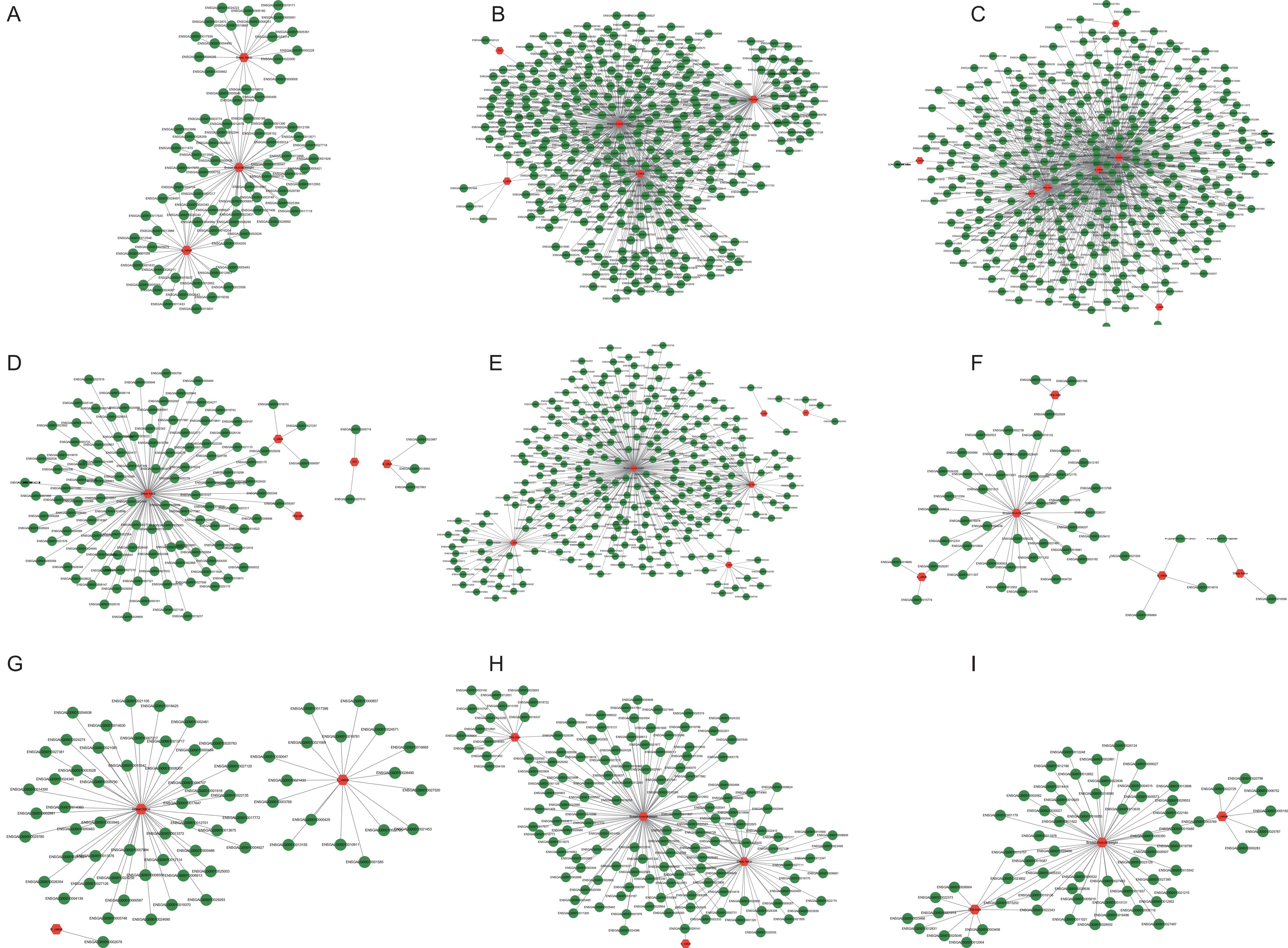
A
B
C
D
E
F
G
H
I
